# Estimating bone marrow adiposity from head MRI and identifying its genetic architecture

**DOI:** 10.7554/eLife.101499

**Published:** 2026-11-04

**Authors:** Tobias Kaufmann, Pål Marius Bjørnstad, Martin Falck, Stener Nerland, Kevin O'Connell, Oleksandr Frei, Ole A Andreassen, Lars T Westlye, Srdjan Djurovic, Timothy Hughes

**Affiliations:** 1 Department of Psychiatry and Psychotherapy, Tübingen Center for Mental Health, University of Tübingen Tübingen Germany; 2 German Center for Mental Health (DZPG), Partner Site Tübingen Tübingen Germany; 3 Norwegian Centre for Mental Disorders Research, Division of Mental Health and Addiction, Oslo University Hospital and Institute of Clinical Medicine, University of Oslo Oslo Norway; 4 Department of Medical Genetics, Oslo University Hospital Oslo Norway; 5 Department of Psychiatric Research, Diakonhjemmet Hospital Oslo Norway; 6 Department of Pharmacy, University of Oslo Oslo Norway; 7 KG Jebsen Centre for Neurodevelopmental Disorders, University of Oslo Oslo Norway; 8 Department of Psychology, University of Oslo Oslo Norway; Maine Medical Center Research Institute United States; University of California, San Francisco United States

**Keywords:** calvarial bone marrow, bone marrow adiposity, artificial neural networks, T1-weighted magnetic resonance imaging, genome-wide association study, mesenchymal stem cell differentiation, Human

## Abstract

Bone marrow (BM) adiposity changes radically through the lifespan, but this phenomenon is poorly characterized and understood in humans. Large datasets of magnetic resonance imaging (MRI) scans of the head have been collected to study the human brain, but also contain unexploited information about other organs. We developed an artificial neural network that localizes calvarial BM in T1-weighted MRI head scans, enabling us to study its composition in several large MRI datasets, and to model sex-dimorphic age trajectories, including the effect of menopause. We revealed high heritability in single-nucleotide polymorphism and twin data, and identified 41 genetic loci significantly associated with the trait, including six sex-specific loci. Integrating mapped genes with existing BM single-cell RNA-sequencing data revealed patterns of adipogenic lineage differentiation and lipid loading. Finally, we identified significant genetic correlations with several human traits, including cognitive ability and Parkinson’s disease, which is intriguing in light of the recently discovered channels that link calvarial BM to the meninges.

## Introduction

Bone marrow (BM) is a tissue located within trabecular bone which produces the cellular components of blood. Beginning in childhood, it undergoes a radical conversion from red to yellow adipose marrow (Moore and Dawson, 1990) which begins in the long bones of the appendicular skeleton, but progresses more slowly in the flat bones of the axial skeleton (Veldhuis-Vlug and Rosen, 2018). By middle-age, BM adipose tissue accounts for 50–70% of total BM volume in healthy humans (Cawthorn and Scheller, 2017). Men have higher bone marrow adiposity (BMA) than women, but post-menopausal women experience a rapid rise in BMA (Griffith et al., 2012). The biological causes and consequences of this phenomenon are poorly understood. It is established that BM fat has a distinct function to white and brown adipose tissue depots (Hardouin et al., 2016; Suchacki et al., 2020), but a precise definition of that function and the evolutionary basis of the sex-dimorphism remain elusive. What is known is that BM adipocytes and bone-forming osteoblasts both result from the bifurcating differentiation of mesenchymal stem cells in the BM niche and this lies at the root of the well-established connection between BMA and osteoporosis (Meunier et al., 1971; Justesen et al., 2001; Rauch et al., 2019). Crucially, BM adipocytes have also been shown to affect the haematopoietic microenvironment (Naveiras et al., 2009).

Magnetic resonance imaging (MRI) scans of the head are primarily thought of as a powerful non-invasive tool for investigating the structure and function of the human brain, but these scans also contain a wealth of imaging information about other organs, such as BM. The calvarial bones contain a BM layer of approximately 600 cm^2^ which is several millimetres thick (Lillie et al., 2016; Law, 1993) and has recently been shown to be connected to the meninges by direct vascular channels through the inner table of the calvarium (Herisson et al., 2018). These channels transport cerebrospinal fluid from the subarachnoid space to the cranial BM via a perivascular route (Pulous et al., 2022; Mazzitelli et al., 2022), and myeloid blood cells travel in the opposite direction (Herisson et al., 2018; Cai et al., 2019; Cugurra et al., 2021). Thus, there is a clear potential for the state of calvarial BM to directly affect the brain. Another specific feature of calvarial BM is that it converts from red to yellow much later in life than the BM of appendicular bones; thus, its composition may be a useful normative marker of ageing.

The gold standard for assessing BM composition is magnetic resonance spectroscopy (MRS), but it is limited by its low spatial resolution, long acquisition time, and need for a predefined volume of interest, making it less well-suited for calvarial BM. Chemical shift imaging techniques offer higher resolution and shorter acquisition time, but such data are not routinely acquired. This has led to a growing interest in using conventional T1-weighted MRI to assess BM composition, with studies reporting robust correlations between BM fat volume and fraction measured with MRS, chemical shift imaging, and T1-weighted pulse sequences (Zhang et al., 2020; Shen et al., 2013; Loevner et al., 2002). Importantly, T1-weighted signal intensity is mainly determined by fat and water content (Loevner et al., 2002; Shah and Hanrahan, 2011; Simonson and Kao, 1992; Cordes et al., 2016), rendering it well-suited to assess BM composition.

Large-scale neuroimaging studies routinely acquire T1-weighted MRI head scans but chiefly focus on the brain. Thus, these datasets present a hitherto unexplored opportunity to address three unresolved aspects of BM biology: First, quantifying in vivo BM composition characteristics at population-scale, including age trajectories and sex-dimorphism, and its relationship to other traits such as bone mineral density (BMD). Second, estimating the heritability and genetic architecture of calvarial BM composition. Third, determining whether the composition of calvarial BM, which envelops the neocortex and has direct channels to it, has detectable effects on the brain.

We developed an artificial neural network-based method for locating calvarial BM in T1-weighted MRI head scans. Since T1-weighted MRI signal intensity is largely determined by fat content, we interpreted the averaged signal intensity as a proxy for calvarial BMA. We conducted extensive validation of the proposed measure using several publicly available MRI resources. We then applied this method to MRI head scans from the *UK Biobank* (Sudlow et al., 2015) to extract information on BMA, its spatial distribution in the calvarium, and to model its main demographic determinants, specifically its relationship with age and sex, including female-specific factors. The size of the sample (*n* = 33,042) enabled us to conduct a well-powered genome-wide analysis (GWAS) to identify gene associations with the proposed measure of BMA and, using a single-cell RNA sequencing (scRNAseq) dataset of BM mesenchymal cells, we gained insight into which genes drive differentiation into the adipogenic lineage and which drive lipid loading. Finally, we investigated the genetic overlap between BMA and 12 brain and body traits.

## Results

### Locating calvarial BM in head MRI scans

**Figure 1.**
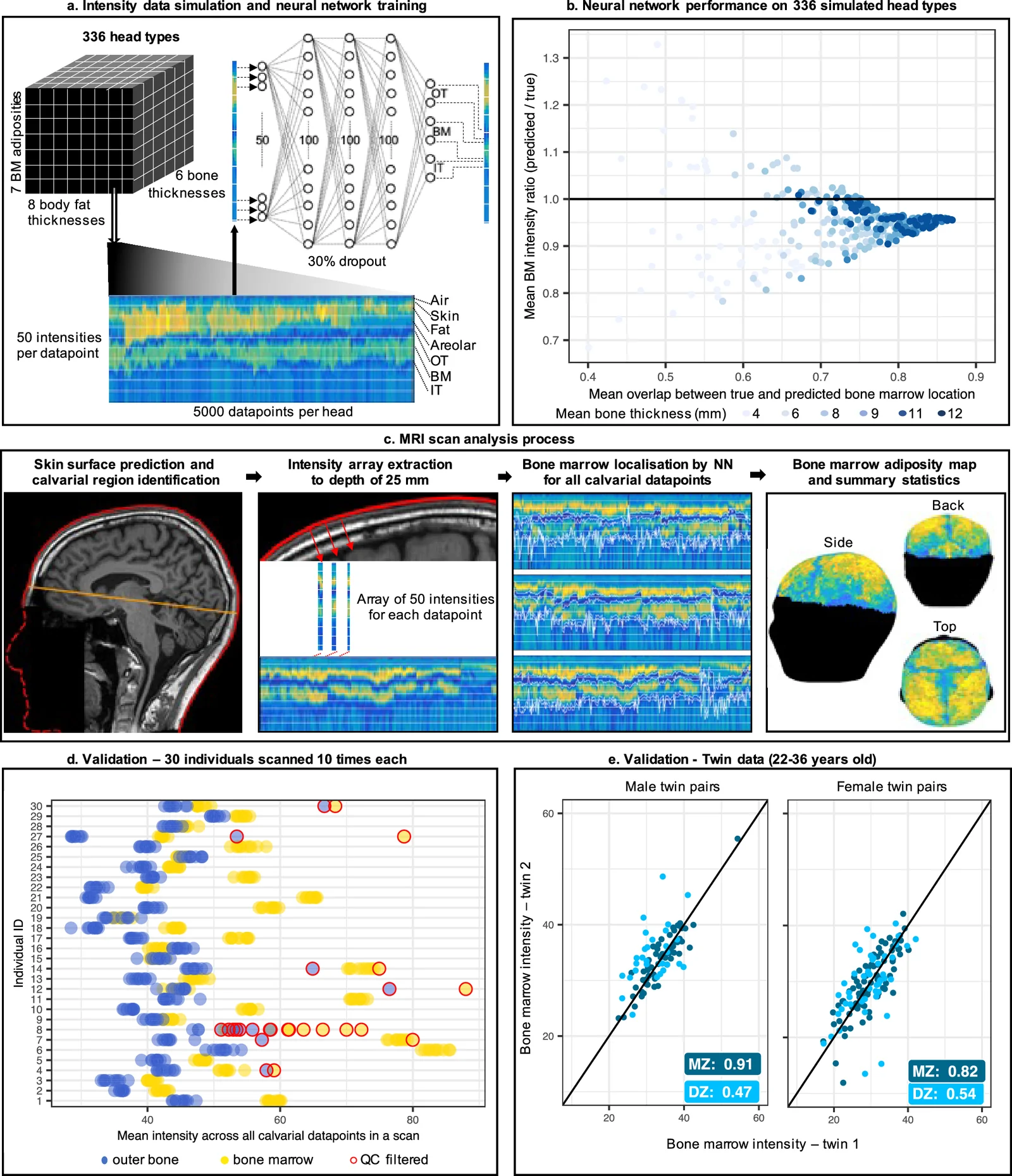
Overview of the artificial neural network training, application, and validation. (**a**) The data used for training the network were generated by simulating 336 different head types (with an example simulation for one head type provided here), where a head type is defined by mean bone thickness, mean BM intensity, and mean body fat thickness. There are 5000 datapoints per head each with 50 intensities per datapoint. OT: outer table, IT: inner table, BM: bone marrow. (**b**) We evaluated the performance of the neural network using two metrics: (1) the overlap between the predicted and the true BM location (defined by the simulation), and (2) the ratio between the predicted signal intensity of the BM and the true intensity of the BM. (**c**) Illustration of the process of extracting the intensity arrays from the MRI scan, locating the BM, and computing its intensity. (**d**) The algorithm was validated on a real dataset consisting of 30 individuals that were scanned 10 times each at 3-day intervals. For each scan, we computed the intensity of the outer bone (blue) and of the BM (yellow), which displayed high concordance across scans of the same individuals. The quality control measures correctly identified scans not producing reliable measures (13 of 300, 11 of which fail due to too few datapoints). (**e**) Application of the algorithm to a cohort of monozygotic and same-sex dizygotic twins. Correlation in BMA between twin pairs in coloured boxes.

Our procedure for locating the BM and measuring its T1-weighted signal intensity involved: (1) identifying the skin surface of the head, (2) identifying the calvarial part of the head using the top of the cerebellum and the accumbens as reference points, (3) for each point of the calvarial surface extracting an intensity array 25 mm into the skull (each layer is 0.5 mm thick), and (4) applying an artificial neural network to identify which layers of the intensity array correspond to BM (Figure 1). This network has 50 input nodes, 3 internal layers, and 6 output nodes (upper and lower bounds of outer table, BM, inner table). We trained the network on simulated data incorporating the natural intra- and inter-individual variation in thickness and intensity of the relevant anatomical structures (Figure 1a, Appendix 1—figure 1). To obtain the BM signal intensity for an individual datapoint of the calvarium, we used the network model to estimate the location of the BM within the datapoint’s intensity array and averaged these BM intensities to get the average BM intensity for that datapoint. Then, we averaged again these datapoint intensities across the calvarium to produce the global BMA measure for the scan.

### Procedure validation on simulated data

We evaluated the performance of the neural network on simulated data using two metrics (Figure 1b): (1) the overlap between the predicted and the true BM location, and (2) the ratio between the predicted intensity of the BM and the true intensity of the BM. The median overlap between the predicted BM and the true BM in the simulated dataset was 0.76 with the lowest value (0.4) obtained in calvaria with a thin bone and the highest value (0.88) obtained in calvaria with thick bone. Poor overlap (below 0.7) was almost only observed in the thinnest bone and is explained by the fact that when the BM part of the bone is only a few layers thick (1 layer = 0.5 mm), an error by one layer will inevitably lead to a substantial fall in overlap. However, this did not result in a corresponding fall in the ratio of the predicted intensity of BM to its true intensity, because the typical BM intensity was only marginally higher than the neighbouring bone intensity. This property of the typical relative intensities of these anatomic structures also explained why the intensity ratio at high overlap is not centred on 1: any misidentification of cortical bone as BM will typically result in an underestimate of true BM intensity (Appendix 1—figure 2). The neural network performed well and intensity ratios were in the range 0.9–1.1 for the vast majority of head types (Appendix 1—figure 3). It was only for heads with thin bone and either very high or very low BM intensity that we observed intensity ratios below or above this range (Appendix 1—figure 3B). Variation in subcutaneous fat thickness had no systematic impact on the performance (Appendix 1—figure 3C).

### Procedure validation on real data and heritability estimate

The procedure also performed well on real data. We first tested a dataset from the *Consortium for Reliability and Reproducibility* (Zuo et al., 2014) consisting of 30 individuals that were scanned 10 times each at 3-day intervals. The scans passing quality control displayed mean intensity of the outer table and BM at the levels that we would expect (Figure 1d): very low variation between scans of the same individual, outer table intensities centred on similar values with low variation across individuals, BM intensity always higher than outer table intensity, and high variation of BM intensity between individuals, but little within individuals. These scans were also inspected visually to verify that structure boundaries were correctly identified. Appendix 1—figure 4 provides examples of network performance in data of individuals with different levels of subcutaneous fat thickness, BMA, and bone thickness. Furthermore, we performed several assessments of the impact of different intensity normalization approaches on the obtained BMA estimates and compared estimates based on T1-weighted images to estimates obtained from T1 relaxation maps derived from a quantitative MRI pulse sequence (MP2RAGE). The BMA measure derived from T1-weighted images significantly correlated with BMA derived from the quantitative T1 relaxation maps and was not prone to differences in standard image normalization procedures (Appendix 1—figures 9–12), supporting the validity of the proposed approach.

The monozygotic (MZ) twin data from the *Human Connectome Project* cohort (Glasser et al., 2016; Figure 1e) provided a second validation of the procedure through the high concordance in mean BM signal intensity between MZ twins, 0.91 in male pairs and 0.82 in female pairs. Further, using the data on the same-sex MZ and DZ twin pairs, we showed that BMA has an estimated broad-sense heritability of 91% in males and 60% in females (Appendix 1—table 1). We estimated a strong influence of environmental factors in females, one of which is highly likely to be estrogen levels as these vary widely between women of child-bearing age, with particularly large changes during pregnancy when estrogen levels are orders of magnitude higher, and the common use of estrogen-based contraceptives.

### The calvarial BMA phenotype

**Figure 2.**
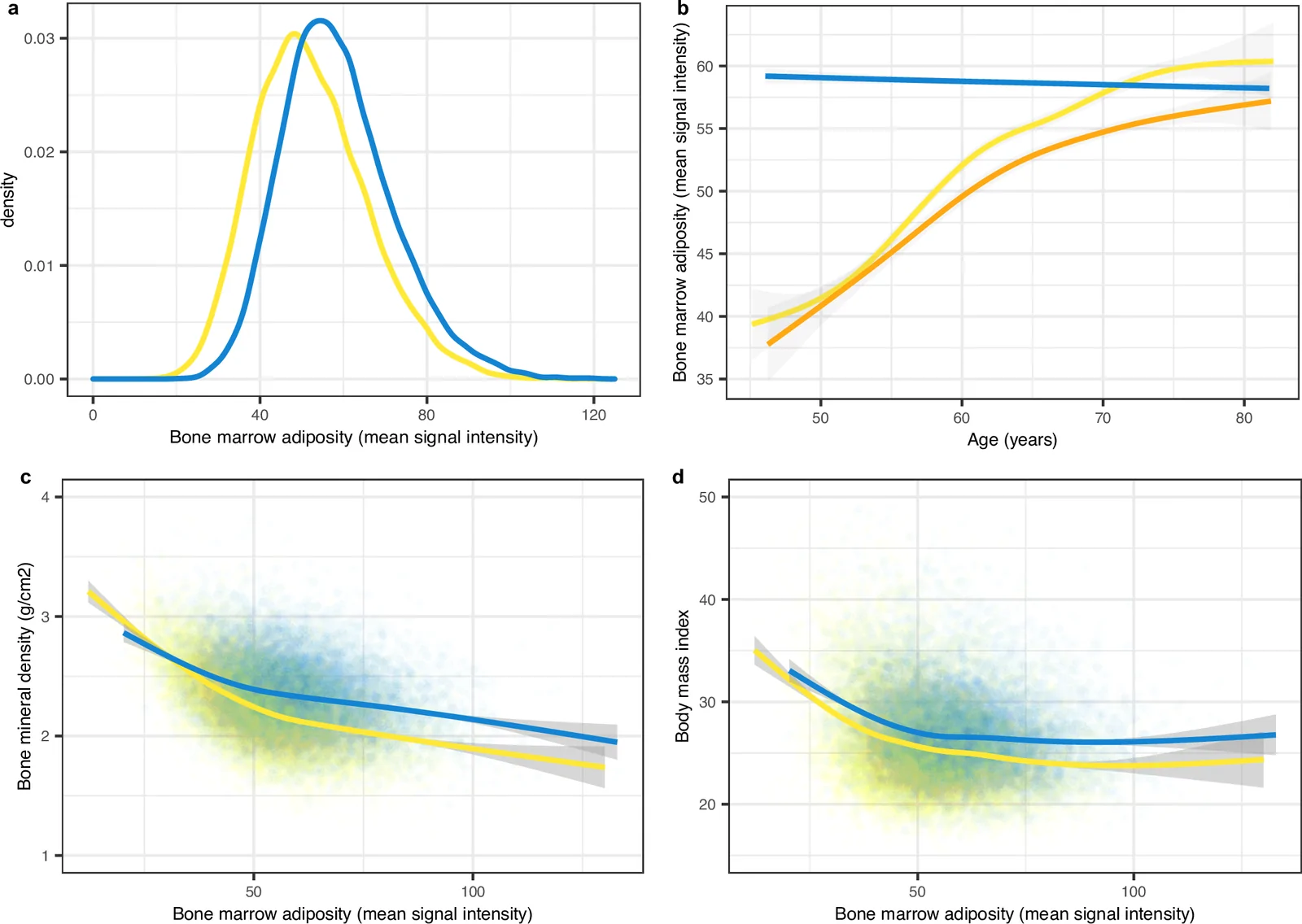
The calvarial BMA phenotype. (**a**) BM intensity distribution in males (blue) and females (yellow). (**b**) BMA age trajectories for males and females. Males in blue and females in yellow (never hormone replacement therapy (HRT)) and orange (ever HRT). The effects of age and HRT on female BMA levels were both statistically significant (p-value <2.0E−16). (**c**) Sex-dimorphic relationship between BMA and calvarial bone mineral density. (**d**) Relationship between BMA and BMI. Fitted lines in (**b**–**d**) were computed using a general additive model, and shading represents 95% confidence intervals.

In the 16,140 male and 16,902 female white British individuals from the *UK Biobank* for which head imaging data is available, we observed that calvarial BMA had strongly sex-dimorphic features. Males had a higher mean BMA than females (Figure 2a) and showed no increase at the group level in BMA levels in the age range covered by the biobank sample (45–82 years), implying that the rise in BMA must have occurred earlier in life (Figure 2b). In females, the BMA level was highly dynamic in this age range, increasing by 50% in the same age interval and thereby reaching male levels at age 70. Hormone replacement therapy (HRT) was used at some point in life by 38% of the women in the study, and this therapeutic intervention slowed the rise in BMA, resulting in average BMA levels for treated women that did not exceed the level of males at age 80.

BM adipocytes and osteoblasts differentiate from the same mesenchymal progenitor cells, and this would appear to drive the known negative correlation between BMA and BMD. We replicated this effect in our data (Figure 2c – 14,262 males and 14,831 females). This relationship is also dimorphic, with females displaying a lower level of calvarial BMD for a given level of BMA. Further, the size of the female BMD deficit increases as BMA levels rise (a similar pattern is observed with BMI in Figure 2d). We quantify the determinants of calvarial BMD by linear regression (Appendix 1—table 2). These regressions confirm the known effects of age and sex, and that the effect of BMA on BMD is about twice as large in females relative to males. Our regression results suggest that most of the effect of HRT on BMD is mediated by its effect on BMA (Appendix 1—table 2) since (1) the statistical significance of HRT as an explanatory variable for BMD disappears when BMA is included as an explanatory variable in the regression and (2) the *R*^2^ of the regression increases by a factor of 7 to 30%.

### Genetic architecture

**Figure 3.**
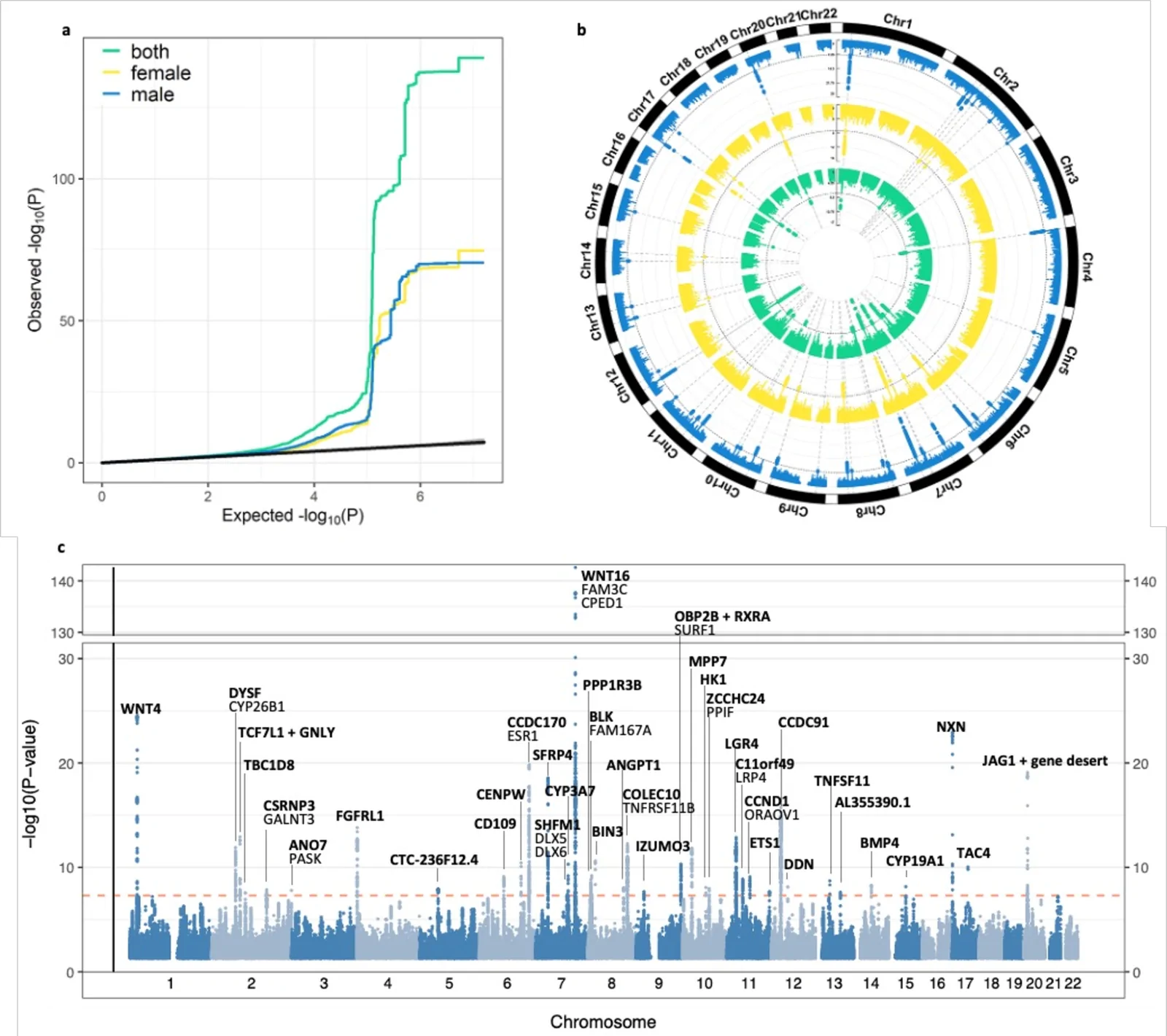
BMA genome-wide significant loci. (**a**) QQ-plot of p-values for the male, female, and joint discovery GWAS. (**b**) Comparison of male (outer), female (middle), and joint (inner) discovery GWAS significant loci. (**c**) Manhattan plot of the 41 genome-wide significant loci in the joint discovery GWAS named after the gene closest to the top lead single-nucleotide polymorphism (SNP) (bold). Other genes in each locus for which genome-wide significant SNPs were eQTLs are listed below the closest gene. Three loci were closely adjacent to another more significant locus and are marked ‘+’. SNPs with p < 0.05 not plotted.

The strong twin-based heritability of BMA emphasized the importance of a genome-wide association study (GWAS) of the trait. We used the *UK Biobank* cohort (Appendix 1—table 3) and performed sex-specific covariate regression prior to separate discovery GWASs on males (*n* = 16,140) and females (n=16,902). We found the male and female GWASs to have low genomic inflation (Figure 3a and Appendix 1—table 4) and the effect sizes to be significantly genetically correlated (Rg = 0.94, p = 6e−27, Figure 3b), allowing us to combine these samples to perform a joint discovery GWAS across males and females (*n* = 33,042). Further, we performed an independent replication GWAS (*n* = 4958) on non-white British males and females.

Single-nucleotide polymorphism (SNP)-based heritability (*h*^2^_SNP_) estimated using linkage disequilibrium score regression confirmed significant heritability of the joint discovery GWAS (*h*^2^_SNP_ = 31.5%) (Appendix 1—table 4). We identified 168 genome-wide significant independent SNPs (p < 5e−8), corresponding to 41 independent genomic loci (Appendix 1—table 5). The replication GWAS also displayed significant heritability (*h*^2^_SNP_ = 28.6%). Out of the 168 significant discovery SNPs, 62% replicated at p < 0.05, and 39% of the 41 lead SNPs replicated at p < 0.05 (Appendix 1—table 6). One locus replicated at genome-wide significance (p < 5e−8, rs3779381, WNT16). Furthermore, 92.7% of the lead SNPs of the discovery sample showed the same effect direction in the replication sample (Appendix 1—table 5, lead SNPs with discordant directions are rs6679981 WNT4, rs117474778 IZUMO3, rs138818878 TNFSF11).

Figure 3c depicts genes mapped from the 41 loci associated with the calvarial BMA. The most significant top lead SNP was located in the WNT16 gene (p = 2.6e−143), which is also the top hit in BMD GWAS (Koller et al., 2013; Morris et al., 2019; Appendix 1—figure 13). The ESR1 locus displayed a notable sex-dimorphic pattern (Appendix 1—figure 14). In females, the top lead SNP overlapped the ESR1 gene, but another lead SNP overlapped the neighbouring CCDC170 gene. In males, the signal in ESR1 was absent, but a genome-wide significant signal was located in the CCDC170 gene, suggesting that the ESR1 gene might not be the only source of an association signal in this locus.

A comparison of the male and female effect sizes of the top lead SNPs of each locus revealed six loci in which there is a more than twofold difference between the sexes (loci 10, 18, 26, 30, 32, and 37 in Appendix 1—table 5). In all cases, it is the male effect size that is larger. Further, each locus is genome-wide significant in males, but not in females, thus suggesting that these loci may be genetic drivers of the sex-dimorphism.

### Gene expression in BM mesenchymal stem cell differentiation

**Figure 4.**
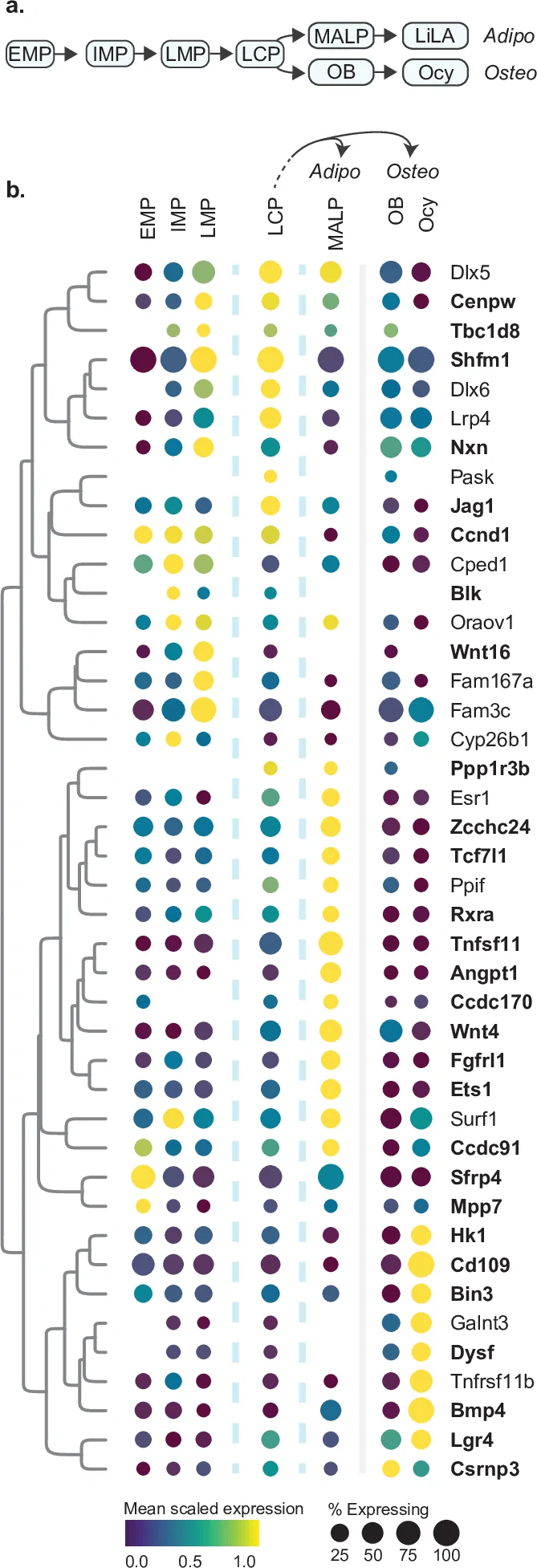
Gene expression in mesenchymal lineage cells of mouse BM. (**a**) The differentiation of BM mesenchymal cells. Early (EMP), intermediate (IMP), and late mesenchymal progenitors (LMP). Lineage committed progenitors (LCP). Marrow adipogenic lineage precursor (MALP). Lipid-laden adipocyte (LiLA). Osteoblast (OB). Osteocyte (Ocy). (**b**) A clustering of the gene expression profiles across cell types. Gene names are for mouse genes (these broadly match human gene names). Of the 40 genes which are closest to the lead single-nucleotide polymorphism (SNP) for each locus, 28 had expression in the scRNAseq data (bold). LiLA expression data was not available in this dataset.

Mesenchymal stem cells of the BM niche commit to either the adipogenic or the osteogenic lineage (Figure 4a), and both the number committing to the adipogenic lineage and their level of lipid-loading influence the total level of BMA. This aspect of BM biology is shared between humans and mice (Tencerova and Kassem, 2016), so we made use of an existing mouse scRNAseq dataset of BM mesenchymal lineage cells (Zhong et al., 2020) to study variation in the expression of BMA-associated genes as cells differentiate (Figure 4b). For any given gene, the percent of cells expressing a gene was relatively stable across different cell types. However, the mean scaled expression varied considerably between differentiation stages, and the clustering of expression profiles revealed that most genes fall into one of the two largest groups of profiles: either genes with their peak of expression at the lineage commitment stage (LMP and LCP) or within the adipogenic lineage (MALP). It was also noteworthy that the genes peaking in MALPs mostly showed low expression prior to that stage. This differed from the ramping up observed in mesenchymal progenitors and LCPs. Only one of the closest genes saw their peak of expression in osteoblasts, but it was unexpected to observe that 6 of the closest genes see a strong peak in osteocytes. It remains to be investigated whether the closest gene might not be the source of the association or whether gene expression in osteocytes may have an influence on BMA.

### Genetic correlation and overlap

We investigated genetic correlation and overlap with 13 traits. We selected body traits (BMD, BMI, waist-to-hip ratio, systolic and diastolic blood pressure, type-2 diabetes, coronary artery disease) that have a logical connection to BMA given the mesenchymal stem cells origin of BM adipocytes and their role in bone, fat, and vasculature (Zhong et al., 2020). For the brain, we selected traits reflecting cognition (general cognitive ability and educational attainment) and disorders that are prevalent in adulthood (insomnia, multiple sclerosis, Parkinson’s disease, Alzheimer’s disease) since it is primarily in adulthood that the adiposity of calvarial BM experiences a substantial change (Figure 2b).

We identified strong negative genetic correlations between BMA and both BMD and BMI, and more subtle global genetic correlations between BMA and Parkinson’s disease, general cognitive ability, and educational attainment (Table 1). However, global genetic correlations lack the ability to assess genetic overlap in the presence of mixed effect directions. So, to better understand the genetic overlap between BMA and other traits of interest, we also applied MiXeR (Frei et al., 2019). MiXeR uses trait polygenicity, defined as the number of trait-influencing variants required to explain 90% of SNP-based heritability, to model the number of shared trait-influencing variants between two traits as well as the correlation within this shared component. MiXeR estimates that 99% of BMA trait-influencing variants are shared with BMD (497 of 500 variants), and that the correlation of this shared component is –0.95, indicating high genetic overlap with discordant effect. The proportion of BMA trait-influencing variants also estimated to influence general cognitive ability (0.57) and educational attainment (0.67) was lower than observed for BMD, but the correlations within their shared components were similarly large (COG = 0.93, EDU = 0.90). These patterns of high overlap and high effect correlation within this overlap are suggestive of vertical pleiotropy, that is a molecular mechanism influencing one trait that, in turn, influences a second trait, such that most of the variants driving the first trait either have the same or the opposite direction of effect on the second trait.

**Table 1.** Genetic correlation and overlap of BMA with other traits. *r*_g_: genetic correlation. *Q*-values are FDR corrected p-values. Genetic overlap: number of causal variants that overlap (i.e. are shared) between BMA and the second trait. Total number of estimated causal variants for a trait (i.e. explaining 90% of single-nucleotide polymorphism (SNP) heritability) is the sum of those unique to trait and overlap (large total is indicative of high polygenicity). NA*: The publicly available summary statistics of the BMI GWAS contained only 3.3 M SNPs; it was thus not possible to apply the MiXeR method to this dataset. Sex-specific genetic correlations, standard errors for causal variant partitioning, and Akaike information criterion values (Appendix 1—table 9). Bold highlighting for: genetic correlation p < 0.01, overlap fraction >0.5, correlation of effect sizes within overlapping >0.5 or<–0.5.

Trait	Genetic correlation (LDSR)			Genetic overlap (MiXeR)						
	**All subjects**			**Partitioning of causal variants** **Mean nb per partition**			**Overlap as fraction of all BMA causal variants**	**Corr. of effect sizes within overlap (**SE)	**Akaike information criterion**	
	***r*_g_**	**p**	***Q***	**Unique to BMA**	**Overlap**	**Unique to trait**			**Best vs min**	**Best vs max**
Bone mineral density	–0.41	**1.5E−22**	**1.8E−21**	3	497	1471	**0.99**	**–0.95** (**0.05**)	13.36	2.47
Body mass index	–0.20	**9.3E−15**	**5.6E−14**	NA*	NA*	NA*	NA*	NA*	NA*	NA*
Waist-to-hip ratio	–0.10	1.2E−02	2.60E−02	63	436	3300	**0.87**	–0.48 (0.16)	5.75	1.30
Blood pressure systolic	–0.02	6.5E−01	6.8E−01	25	475	3921	**0.95**	–0.25 (0.05)	45.31	4.85
Blood pressure diastolic	–0.03	3.4E−01	4.1E−01	27	473	3541	**0.95**	–0.21 (0.04)	58.37	2.77
Coronary artery disease	–0.10	3.6E−02	7.2E−02	275	225	1140	0.45	–0.40 (0.11)	8.53	24.01
Type-2 diabetes	–0.07	6.5E−02	1.1E−01	361	139	752	0.28	**–0.69** (**0.16**)	8.75	77.44
Insomnia	–0.05	2.1E−01	2.8E−01	248	252	8868	0.50	–0.45 (0.32)	2.49	3.57
Multiple sclerosis	–0.02	6.8E−01	6.8E−01	474	26	86	0.05	0.45 (0.14)	5.82	42.60
Parkinson’s disease	–0.15	**1.7E−03**	**4.1E−03**	432	68	844	0.14	**–0.75** (**0.14**)	1.55	41.25
Alzheimer’s disease	–0.13	9.2E−02	1.4E−01	485	15	52	0.03	**–0.61** (**0.24**)	10.90	37.84
General cognitive ability	0.09	**1.1E−03**	**3.3E−03**	217	283	10,802	**0.57**	**0.93** (**0.12**)	1.47	8.73
Educational attainment	0.10	**9.9E−05**	**4.0E−04**	166	334	12,855	**0.67**	**0.90** (**0.11**)	2.41	9.45

In Parkinson’s disease, where we also estimated statistically significant genetic correlation with BMA, MiXeR estimates a strong negative correlation of effect sizes, but this is within a much lower percentage of BMA trait-associated variants (14%). Similarly, BMA displayed a high correlation of effect sizes (–0.69) with type-2 diabetes but within a moderate overlap, and a low correlation of effect sizes with both systolic and diastolic blood pressure, but with an overlap of 95%. These patterns are more indicative of horizontal pleiotropy (i.e. molecular mechanisms influencing multiple traits) than vertical pleiotropy.

## Discussion

The development of a method for locating BM in MRI head scans enabled us to characterize calvarial BMA in several datasets and to quantify, in vivo and at population-scale, BMA’s age trajectory, sex-dimorphism, and relationship to BMD. We focused on BM adiposity rather than volume, as this trait was considered more likely to be influencing other traits of interest, such as the blood (haematopoiesis), bone (osteoporosis), and brain health (through transcortical channels). Indeed, through a well-powered GWAS, we identified the main genetic drivers of this phenotype and we observed significant genetic correlation and overlap with several traits, including Parkinson’s disease and general cognitive ability.

### Estimating BMA in MRI head scans

BMA can be quantified in whole-body scans (Morris et al., 2022), but calvarial BM has a number of features which make it particularly well-suited to the study of BMA. First, conversion to yellow BM is slower in the flat bones (Chan et al., 2016), such that sex-dimorphic trajectories are observable in middle-aged cohorts. Second, there are many large collections of T1-weighted MRI scans from which calvarial BMA can potentially be extracted and, although T1-weighted pulse sequences are not specifically designed for BMA quantification (Karampinos et al., 2018), this enables analyses that have not previously been possible, such as GWAS and accurate estimation of lifespan sex differences. Finally, it has a simple ‘sandwiched’ structure that we were able to model and then generate simulated calvaria which we use as training data for an artificial neural network. Our validation procedures demonstrate that this automated BM location procedure delivers good performance on real data, even though it was trained on simulated data and analyses each datapoint independently of all others. A more sophisticated model (Zopes et al., 2021; Çiçek et al., 2016), trained directly on annotated real data, may raise performance further, for example by improving the prediction of the lower bound of the BM. However, this would require a substantial manual annotation effort and would face the difficult challenge of distinguishing low-adiposity BM from calvarial bone. Both these issues are circumvented by our approach.

### The calvarial BMA and BMD phenotypes

The sex-dimorphic nature of BM adiposity is qualitatively well-established (Griffith et al., 2012), but has not previously been quantified in a large human cohort. Males have a high and stable average level of BMA over the covered age range (45–82 years of age), indicating that the dynamic male phase occurs earlier in life. The youngest females in our sample have a low level which ramps at a constant rate from the beginning to the end of the range with HRT significantly reducing the rate of increase. Interestingly, however, by the age of 70 (for women not using HRT), female BMA has risen to the same level as males. It appears highly likely that the menopause and the accompanying drop in natural estrogen levels is part of what drives the rise in BMA levels and that estrogen treatment can delay the rise. We emphasize that these trajectories are for calvarial BM and are most probably different in profile and levels to other BM, since it is known that axial flat bones convert later and perhaps less completely than the appendicular long bones. However, the nature of the sex-dimorphism and the effect of estrogen is likely shared with other BM.

Women at risk of osteoporosis are often prescribed HRT in order to improve BMD. Our regression results (Appendix 1—table 2) indicate that the effect of HRT on BMD is mostly mediated by its effect on BMA. Further, we suspect that the effect of BMA on BMD may be underestimated given that the prescription of HRT is biased towards women with low BMD and high BMA. Future studies, including information on why HRT was prescribed and for how long, may use our BMA quantification method to further detail this important relationship.

### BMA heritability estimates

Our findings showed that male SNP heritability (43%) is half the broad-sense heritability (91%). In females, SNP heritability is only 23% which is low relative to men and relative to the broad-sense heritability of 60%. Since the vast majority of women in the *UK Biobank* sample have probably not reached their ultimate BMA levels, we suspect that the genetic effects on the phenotype may be underestimated in this female GWAS sample. Despite these features of the sample and its relatively modest size, we do observe good genetic correlation between the sexes of the discovery sample (Figure 3b) and cross-ethnic replication in a limited sample of 4958 individuals. It should be noted that this is not equivalent to replication in an independent cohort of similar ancestry and is expected to show reduced SNP-level reproducibility because of differences in sample size and genetic background. Based on these GWAS results, MiXeR estimates that BMA has approximately 500 causal variants, indicating low polygenicity relative to a typical polygenic trait such as BMD, which we estimated to have approximately two thousand causal variants.

The high broad-sense heritability estimates indicate that environmental effects on BMA are limited relative to genetics. However, a limitation of our broad-sense heritability estimates is that the age range of the twin dataset is much younger than the *UK Biobank* dataset from which we compute SNP heritability. This is particularly relevant for females, first because fluctuations in estrogen levels are substantial during child-bearing age due to pregnancy and contraception, and second because females only reach their ultimate BMA level between the ages of 70 and 80 (the upper end of the age range). Both the broad-sense and SNP heritability estimates should therefore be interpreted with caution for females.

### Genetic correlation and overlap with other traits

We selected seven body and six brain traits for which we computed genetic correlation and obtained significant results for BMD, BMI, Parkinson’s disease, general cognitive ability, and educational attainment. Genetic correlation has two limitations. First, it tends to underestimate the true correlation in the case of mixed effects and, second, it provides no indication of whether the relationship between the traits is driven by horizontal or by vertical pleiotropy. MiXeR estimates the causal variants for each trait and thereby can estimate the overlapping fraction as well as the correlation of effect within this set. A high overlap of BMA causal variants with those of the other trait and a high correlation of effects within this set is suggestive of causal effect (vertical pleiotropy). Only 3 of the 12 traits displayed a clear combination of such features. One of these is BMD, which can be considered as a positive control since the BMA and BMD traits are known to be negatively correlated and the bifurcating differentiation of mesenchymal stem cells provides a causal cellular mechanism for this relationship between traits. The other two traits are educational attainment and general cognitive ability. Since these are two highly correlated traits (Savage et al., 2018), a high overlap and correlation of genetic effects for both traits with BMA may be consistent with the hypothesis that BMA could have a causal effect on cognition (Table 1).

### GWAS results and differentiation of mesenchymal progenitors

Previous studies of mesenchymal cell differentiation in animal and cell models have pointed towards the Wnt/β-catenin signalling pathway being an important determinant of BMA (Tencerova and Kassem, 2016). Our large-scale phenotyping of BMA in a human cohort enabled us to perform a hypothesis-free quantification of its genetic architecture, which confirmed the strong effect of genes related to this pathway, including WNT16 (Shen et al., 2018), WNT4 (Zhang et al., 2021), and NXN (Funato and Miki, 2007). Further, we found associations in genes (BMP4 [Bowers et al., 2006; Bowers and Lane, 2007], DLX5 [Rauch et al., 2019; Kim et al., 2004], and LGR4 [Sun et al., 2019]) or close paralogs of genes (LRP4/LRP5 [Qiu et al., 2007], SFRP4/SFRP1 [Taipaleenmäki et al., 2011]) that were known to specifically influence adiposity. We also discovered and quantified the effect of many interesting novel loci, such as FGFRL1, which has a pleiotropic role in hypertension resistance and bone growth in giraffes (Liu et al., 2021). The strong effects of the CCDC170/ESR1 and WNT16 loci (Appendix 1—table 5) were not unexpected given previous results from the BMD GWAS (Morris et al., 2019), but the pattern of the genetic signal illustrates the importance of fine mapping for these and other loci (Appendix 1—figures 13 and 14).

Recent scRNAseq studies of the BM niche have provided a comprehensive insight into BM cell types and the changes in gene expression during differentiation (Baryawno et al., 2019; Tikhonova et al., 2019; Dolgalev and Tikhonova, 2021). Zhong et al., 2020 focused specifically on mesenchymal lineage cells and produced a detailed transcriptomic profile of progenitor maturation and bifurcating differentiation into adipogenic and osteogenic lineages. Interestingly, they identify a new non-proliferative cell type in the adipogenic lineage that does not contain lipid droplets (MALP: marrow adipogenic lineage precursors). In our clustering of GWAS genes according to their scRNAseq transcriptomic profiles, we find 17 genes that ramp expression in mesenchymal progenitors with the peak of expression occurring in LMP or LCP, and 16 genes with very low expression in mesenchymal progenitors and a distinct peak at the MALP stage. Since BMA is primarily determined by two potentially asynchronous processes, the number of adipocytes and the level of lipid-loading, this suggests that the first set (including WNT16) controls the bifurcation between the two main lineages, while the second set (including ESR1) controls MALPs further differentiation into lipid-laden adipocytes.

MALPs outnumber lipid-laden adipocytes by orders of magnitude in young mice and exist as pericytes and stromal cells that form a ubiquitous 3D network inside the marrow cavity (Zhong et al., 2020). Evidence points to this network playing pivotal roles in maintaining marrow vasculature (Zhong et al., 2020), suppressing osteogenic differentiation (Pino et al., 2016), and probably also influencing haematopoiesis (Naveiras et al., 2009). We tentatively speculate that calvarial MALPs may be involved in sensing perivascular flows of CSF from the meninges to the BM and in influencing the BM’s haematopoietic response, and that this might be the basis of the observed genetic overlap between BMA and some cerebral traits. Beyond these perivascular pathways, established cranial venous anatomy provides other possible interfaces between calvarial marrow and the intracranial compartment, particularly through diploic veins that communicate with meningeal veins and dural venous sinuses.

### Limitations and future validations

Several previous studies have used T1-weighted MRI to study BM composition (Okada et al., 1989; Kimura et al., 1990; Shen et al., 2007). Validation studies have reported correlations of 0.99 with BM fat volume and of 0.71 with BM fat fraction measured with the IDEAL technique in the tibia of children (Zhang et al., 2020). Similarly, BM fat volume estimated with T1-weighted images has shown strong correlations with MRS (*r* = 0.88) and the Dixon method (*r* = 0.79) for vertebrae and with the Dixon method for femoral neck (*r* = 0.86) in menopausal women (Shen et al., 2013). A score based on T1-weighted signal intensities in the vertebrae was found to be a promising marker for osteoporosis and correlated with BMD measured with DEXA (*r* = −0.68) (Bandirali et al., 2015). While most prior studies have focused on non-cranial BM, one study found that T1-weighted signal intensity in the calvarium was a sensitive (93%) and specific (86%) marker for infiltrative processes including lymphoma, leukaemia and chronic anaemia known to affect BM composition (Loevner et al., 2002).

Whereas prior studies have relied on manual grading and selection of segmentation thresholds and regions of interest, our approach automatically locates calvarial BM. This improves repeatability and allows for deployment in large datasets such as the UK Biobank. Although the main determinant of the T1-weighted signal intensity is fat, making it well-suited to assess BMA, weighted MRI pulse sequences present notable challenges. Importantly, T1-weighted signal intensity is influenced by scanner-related variation (Wiltgen et al., 2024).

To ensure between-subject comparability, we used the intensity normalized nu.mgz volume output by FreeSurfer. We validated this approach through comparison with well-established intensity normalization methods: Kernel Density Estimation (KDE), WhiteStripe (WS), Gaussian Mixture Model (GMM), Fuzzy *C*-Means (FCM), and *Z*-score normalization (ZS). We found the highest test–retest reliability with our approach (Appendix 1—figure 9), and, together with KDE (based on reference signal intensity in WM), the highest correlation with quantitative T1 relaxation maps (Appendix 1—figure 10). Together with the significant correlations with BMD (Appendix 1—figure 11), as measured with DEXA, and significant effects of osteoporosis status (Appendix 1—figure 12), these results demonstrate the biological relevance of the proposed measure of BM composition.

Nevertheless, we recognize the need for further validation to disentangle the relative contributions of adiposity and other features of BM composition. Ideally, a sufficiently powered data set with both T1-weighted imaging and quantitative multi-echo fat-water imaging of the calvarium would be needed, yet to the best of our knowledge no such data set is available to date. Respective sequences are available, but may need to be fine-tuned to match the properties of the thin calvarium. Given the sexual dimorphism in BMA reported in our work, it appears best to focus recruitment of the initial validation sample on women in the menopausal transition period so as to ensure sufficient signal variability in the data. Until such data is acquired and analysed, caution is still warranted on the biological interpretation of measures based on T1-weighted images. However, given the plausible biological findings in our study (sex-dimorphic age trajectories mapping known BMA biology, discovery of genes previously identified through cellular experiments as critical to adipocyte biology), we are confident that the benefits of exploiting the existing large samples of T1-weighted images outweigh the limitations. Indeed, future studies can build on our developments to further validate the proposed measure of BM composition and to study its effect on bone, blood, and brain.

## Materials and methods

### Experimental design

This study had several sequential subgoals: (1) obtain data on BM composition from head MRI scans, (2) determine the sex-dimorphic age trajectories, (3) identify the genes associated with this trait, (4) determine which of these genes control the bifurcating (osteogenic/adipogenic) differentiation and which control lipid loading, and (5) investigate whether the BM composition trait has genetic overlap with other related body or brain traits. Each of these study components requires specific methodology and several data sources are used. These are described separately in the following subsections.

### Cohorts

#### Hangzhou Normal University (HNU) data as part of the Consortium for Reliability and Reproducibility (Zuo et al., 2014)

We accessed the openly available test–retest reliability dataset from http://dx.doi.org/10.15387/fcp_indi.corr.hnu1. In this study, T1 MRI was acquired on a 3T GE Discovery MR750 scanner using an 8-channel head coil with a repetition time of 8.06 ms, 8° flip angle, and 250 mm field of view at a resolution of 1 × 1 × 1 mm. We included all available data from this sample, specifically data from 30 healthy adults (50% female) aged 20–30 years (mean: 24.4, SD: 2.4 years) that were scanned ten times across 1 month, one scan every 3 days.

#### MICA-MICs data (Royer et al., 2021)

We accessed the openly available multimodal dataset from https://portal.conp.ca/dataset?id=projects/mica-mics. In this study, MRI data was acquired on a 3 T Siemens Magnetom Prisma-Fit equipped with a 64-channel head coil. T1 MRI was acquired with a 3D magnetization-prepared rapid gradient-echo sequence (MPRAGE; 0.8 mm isotropic voxels, matrix = 320 × 320, 224 sagittal slices, TR = 2300 ms, TE = 3.14 ms, TI = 900 ms, flip angle = 9°, iPAT = 2, partial Fourier = 6/8). Quantitative T1 MRI was acquired with a 3D-MP2RAGE sequence (0.8 mm isotropic voxels, 240 sagittal slices, TR = 5000 ms, TE = 2.9 ms, TI 1=ld0 ms, T1 2 = 2830 ms, flip angle 1=4°, flip angle 2 = 5°, iPAT = 3, bandwidth = 270 Hz/px, echo spacing = 7.2 ms, partial Fourier = 6/8).

#### Human Connectome Project data (Van Essen et al., 2013)

We accessed openly available data from https://db.humanconnectome.org. In this study, T1 MRI was acquired on a customized 3T Siemens Skyra scanner with a repetition time of 2400ms, 8° flip angle, 224 mm field of view, and a voxel size of 0.7 mm. We included data from 430 twins from this sample, including 174 males (60 dizygotic and 114 monozygotic, age range 22–34) and 256 females (96 dizygotic and 160 monozygotic, age range 26–36).

#### UK Biobank (Sudlow et al., 2015)

We accessed data from the UK Biobank study resource (access code 27412; https://www.ukbiobank.ac.uk). In this study, T1 MRI data was acquired on three identical 3T Siemens Skyra scanners with a repetition time of 2000ms, 8° flip angle, 256 mm field of view, and a resolution of 1 × 1 × 1 mm. We split the available sample into a discovery set comprising 33,042 white British individuals (51% females) aged 45–82 years (mean 64.9, SD 7.5 years), and an independent replication set comprising all other individuals (*N* = 4958, 52% female, age mean 62.9, SD 7.7, range 45–81 years). We also obtained head BMD measurements produced by a DXA system (code 23226-2.0), body mass index (code 21001-2.0), and data on whether women ever used hormone-replacement therapy (code 2814-2.0). BMD data was only available for 29,093 of the samples for which MRI head scans were available.

### FreeSurfer preprocessing and extraction of datapoint intensities

We processed all T1 MRI data using the standard recon-all pipeline in FreeSurfer 5.3.0. T1 intensities were extracted from the nu image, which is corrected for intensity non-uniformities and intensity normalized via scaling. Next, we fed the intensity-normalized volumes derived via recon-all into the FreeSurfer mri_watershed program to delineate the surfaces of the brain, skull, and skin. We performed a stepwise extraction of intensities from layers following the shape of the outer skin, walking inward into the skull in steps of 0.5 mm up to 25 mm inward, which produces an intensity array of length 50 for each datapoint. Since watershed delineates the skin around the entire head, we disregarded parts that are not directly on top of the cortex. For this, we identified the coordinates of the upper part of the cerebellum and the accumbens from the individual level FreeSurfer aseg parcellation and defined a plane in 3D space that intersected these two points, disregarding all datapoints in coordinates below the plane. The resulting stream of layerwise intensities therefore comprised data on top of the cortex and was fed one-by-one into an artificial neural network model to identify and define the borders of the BM for that datapoint. See supplementary materials for more detail on this workflow, including software versions.

### Intensity array data simulation

In order to train the neural network model, we generated a large synthetic dataset of intensity arrays, with known boundaries between anatomical structures, by simulating the thickness and intensity of the different structures located between the outer skin and the sub-arachnoid space. The simulation incorporated the following real-world complexities:

1. Different anatomical architectures (skin, subcutaneous fat, aponeurosis, outer table, BM, inner table, dura mater, arachnoid space), including when a structure is not present throughout the calvarium.
2. Variation in thickness and intensity between vertices (on the same calvarium).
3. A wide variety of different calvarium types with different combinations of levels of BM adiposity, bone thickness, and subcutaneous adiposity.
4. ‘Blurring’ between anatomic structures was present in real data and was modelled in our simulations:
  - Blurring that occurred because of the voxel resolution of the MRI scanner relative to the underlying structures.
  - Blurring that occurred because the layers for a datapoint usually do not match with voxel boundaries.

The parameters used in the simulation (primarily mean and variance of both thickness and intensity of each of the different anatomical structures within a calvarium) were estimated by reviewing the MRI scans of a wide variety of real subjects from the MRI scan collections included in this study. We simulated 336 different calvaria with the following mean characteristics: 7 BM intensities (40, 50, 70, 90, 110, 140, and 170) × 6 bone thicknesses (4, 6, 8, 9, 11, and 12 mm) × 8 body fat thicknesses (0, 1, 2, 3, 4, 6, 9, and 12 mm) and for each calvarium we generated 5000 intensity arrays. In the iterative process of simulating data, training the network, and testing its performance on real data, we observed that incorporating the characteristics of extreme head types in the simulation parameters was critical to obtaining good performance on real data. Fine-tuning of model hyperparameters played a much lesser role. The R code and all parameters are openly available (see code availability).

### Artificial neural network architecture and training

The network is a feed-forward fully connected network. There are 50 input nodes (for the 50 intensities of a datapoint), three internal layers with 100 nodes each (ReLU activation function), and six output nodes (upper and lower bound for each of outer table, BM, and inner table). The model was implemented in the Keras framework on top of the TensorFlow backend, and the training of the model was performed in Google Colab (Google, 2026). All vertices from all calvaria were randomly shuffled, and then two thirds of the data were used for training the model, and one third was used as the validation set to measure the performance. The loss function, defined as the sum of the mean squared errors of the six outputs, was minimized using the Adam algorithm (Kingma and Ba, 2014), with a learning rate of 0.1 and a batch size of 8192. We used dropout to regularize the model (Srivastava et al., 2014), with a 30% dropout rate in all the hidden layers. The model is trained over 300 epochs. The performance of the network plateaus before 300 epochs (Appendix 1—figure 1), suggesting that the optimization has converged on the best parameters for this model architecture.

To compute the overlap of the true and the predicted intervals, the predictions were first rounded to integers, so they refer to specific layers in the intensity vector. Then the overlap is defined as the number of layers that are included in both the predicted and the true interval, divided by the number of layers in the longest of the true and the predicted interval. For each datapoint of a scan, we computed the mean BM intensity from the part of the intensity array predicted to be BM and then computed the mean and standard deviation across all datapoints in the scan. We did the same for the outer and inner table. The Python code is openly available (see code availability).

### Additional validation of the BMA estimation approach

In the BMA estimation pipeline, we used intensity normalized T1 images generated in the standard recon-all processing stream of FreeSurfer. To assess the effect of intensity normalization, we estimated BMA with five additional frequently used intensity normalization methods (Reinhold et al., 2019). We calculated test–retest reliability (Appendix 1—figure 9) and, in a dataset where quantitative T1 data was available (Royer et al., 2021), compared BMA estimates derived from quantitative T1 with our semi-quantitative approach, as well as each of the five additional intensity normalization methods (Appendix 1—figure 10). Finally, we further assessed the semi-quantitative nature of our approach by comparing outer-bone intensity with BMD as measured by DEXA scans (Appendix 1—figure 11).

### ANN quality control measures

We performed the BM location procedure on the intensity data from 42,068 heads in the UK Biobank. 420 of these produced no output and 2,234 produced a calvarium with less than 5,000 vertices most likely related to poor image quality (Appendix 1—figure 5), leaving 39,414 samples.

We used two additional QC metrics to filter out calvaria where BM location was likely to have failed. First, we set an upper limit of 30 on the standard deviation of the intensity of the outer table as scans with higher values were clear outliers and were probably cases where the location of both outer table and BM has failed (Appendix 1—figure 6). Second, for each calvarium, we computed the Mahalanobis distance for all vertices in the two dimensions ‘first layer of the BM’ and ‘BM intensity’ (Appendix 1—figure 7). By manual inspection we found that datapoints with MD >25 often had errors in BM layer identification, typically where the network had erroneously predicted a higher and more intense layer to be the BM. We considered a calvarium as failing this QC criterion if more than 0.5% of vertices have MD >25. This criterion is very strict as errors on only 0.5% of datapoints in a calvarium would not significantly affect the average BM intensity for a calvarium. However, we wanted the data to be usable to generate individual calvarial BMA maps and were only willing to tolerate a very low percentage of datapoints with errors in each calvarium. These two additional QC filters were complementary (Appendix 1—figure 8) and resulted in a final number of 39,207 scans passing QC.

### Twin heritability estimates

We used the monozygotic and same sex dizygotic twins from the *Human Connectome Project* dataset to fit the NACE model using the twinlm function of the mets package (Holst et al., 2016) in R (Appendix 1—table 1).

### GWAS methods

We followed standard quality control procedures for preparing UK Biobank genetic data. Specifically, we removed individuals missing more than 1% of genotyping data, and SNPs missing in more than 5% of individuals or with a minor allele frequency below 0.01, yielding approximately 7.9 M SNPs. We split the available sample into a white British discovery set and a non-white replication set, as detailed under *cohorts*. Within each of these samples, we performed sex-specific covariate regression prior to genome-wide association analysis (GWAS). For males, we residualized our estimates of BM adiposity from age (using orthogonal polynomials of degree 2), scanning site, and the first 20 genetic principal components for population level stratification. For females, we applied the same model yet also controlling for hormonal replacement therapy as this is known to affect BMA (Syed et al., 2008) (binary: ever used HRT therapy yes/no).

The residuals were fed into GWAS using plink (version 2) (Purcell et al., 2007). Using the discovery sample, we first performed a GWAS in females only (*N* = 16,902), and a GWAS in males only (*N* = 16,140). After validating their significant genetic correlation (Rg = 0.94, p = 6e−27), we performed a GWAS in the full discovery sample, merging the residuals from the female model with the residuals from the male model. Likewise, we performed a GWAS in the replication sample merging the residuals from a male and a female model in a joint GWAS (no sex-specific GWAS performed due to power in this sample). The resulting summary statistics were standardized using the python_convert toolbox (https://github.com/precimed/python_convert, Frei and Roelfs, 2024), removing the MHC region in chromosome 6 between base pairs 26M and 34M before feeding them into genetic correlation analysis. GWAS summary statistics are available on the FUMA website (see data availability).

GWAS results were post-processed using the Functional Mapping and Annotation of Genome-Wide Association Studies (FUMA) tool (Watanabe et al., 2017) to define genomic loci, significant independent SNPs, lead SNPs, and top lead SNPs. We used default settings which were N = NA, Ncol = N, exMHC = 1, MHCopt = annot, extMHC = NA, ensembl = v92, genetype = protein_coding, leadP = 5e−8, gwasP = 0.05, r2 = 0.6, r2_2 = 0.1, refpanel = 1 KG/Phase3, pop = EUR, MAF = 0, refSNPs = 1, mergeDist = 250. FUMA was also used to perform an eQTL analysis of the GWAS results using the GTEX v8 expression dataset. Genes, which were not the closest gene to the top lead SNP of a locus, but for which genome-wide significant SNPs were strong eQTLs, were annotated in the Manhattan plot (Figure 3).

### Single-cell data and analysis

Single-cell RNAseq data of BM mesenchymal lineage cells (Zhong et al., 2020) was downloaded from the Single Cell Portal (here) and used to create a Seurat 4.0 object in R-studio (count, embedding, and meta-data). This was an integrated dataset of 7585 cells that contains endosteal Td+ BM cells from 1-month-old (1M) and 1.5-month-old (1.5M) male Col2:Td mice. We did not modify the cell clustering or nomenclature, other than excluding two chondrocyte clusters as chondrocytes originate from the resting zone progenitors, but not BM MSCs (Zhong et al., 2020). The dataset does not include lipid-laden adipocytes presumably due to the difficulty in analysing these large and fatty cells using this method.

We searched the data from Zhong et al. for the 54 genes displayed in the Manhattan plot (Figure 3). Human gene names were translated to mouse gene names and, if no similar name was identified, we searched for orthologs. For 3 of the 54 human genes, we could not identify a mouse gene and, for an additional 9 genes, the mouse gene had no expression in the scRNAseq data (Appendix 1—tables 7 and 8). Cluster gene expression was plotted as a dotplot (ggplot2) and patterns in gene expression were grouped by alignment to a dendrogram (ggtree) based on Euclidean distance. The expression of each gene was scaled by the gene’s mean expression across clusters (i.e. cell types). Zero expression dots were removed.

### GWAS downstream analysis and genetic architecture

We estimated SNP heritability and genomic inflation of the resulting GWAS summary statistics using LD-score regression (Bulik-Sullivan et al., 2015). The same tool was used for estimating genetic correlations with BMD (Morris et al., 2019), body mass index (Yengo et al., 2018), waist-to-hip ratio (Shungin et al., 2015), blood pressure (Evangelou et al., 2018) (systolic and diastolic), coronary artery disease (Nikpay et al., 2015), type-2 diabetes (Mahajan et al., 2018), multiple sclerosis (Patsopoulos et al., 2019), Parkinson’s disease (Nalls et al., 2019), Alzheimer’s disease (Wightman et al., 2021), general cognitive ability (Savage et al., 2018), educational attainment (Lee et al., 2018), and insomnia (Jansen et al., 2019).

To estimate genetic overlap between pairs of traits, we used bivariate MiXeR v1.3 (https://github.com/precimed/mixer; Frei and Karthikeyan, 2025). MiXeR (Frei et al., 2019) uses a Gaussian causal mixture model to estimate the total number of trait-influencing variants that explains 90% of SNP heritability (i.e. polygenicity). In this case, a trait-influencing variant is a common variant with a direct effect on the trait of interest excluding effects due to LD. Parameters are estimated with 20 iterations of the mixture models and the mean of each estimate is quantified along with the standard deviation over the 20 iterations. Given two traits of interest, MiXeR models the trait-influencing variants unique to each trait (non-overlapping) as well as the number of shared trait-influencing variants (overlapping). To assess model fit, MiXeR employs the difference in Akaike information criterion between the MiXeR model and an infinitesimal model with a positive value indicative of good model fit.

### Statistical analysis

All statistical analyses are described in the relevant subsections above.

### Code availability

All code and software needed to generate the results are available as part of public resources: LD score regression (https://github.com/bulik/ldsc, Bulik-Sullivan et al., 2026), FUMA (https://fuma.ctglab.nl/), MiXeR (https://github.com/precimed/mixer, Frei and Karthikeyan, 2025), and BM location (simulation, training, and model: https://github.com/comgen/boneMarrowLocalisationT1wMRI, copy archived at Hughes, 2024).

## Figures and Tables

**Appendix 1—figure 1.**
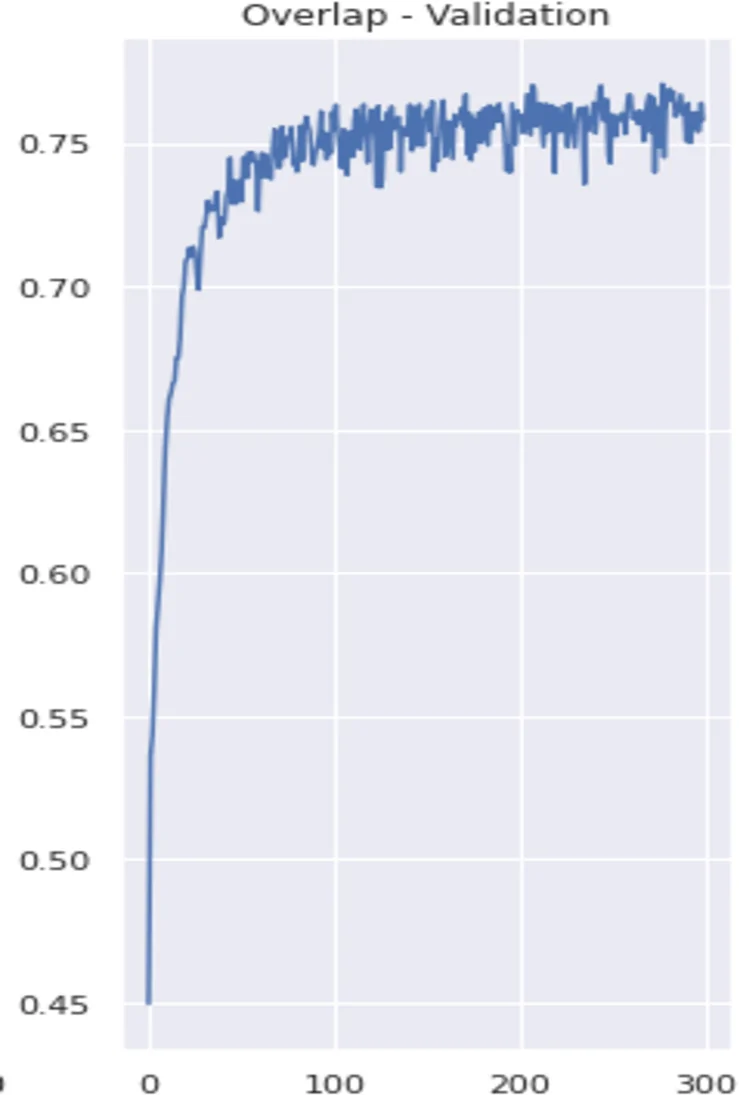
Convergence of the artificial neural network before 300 epochs.

**Appendix 1—figure 2.**
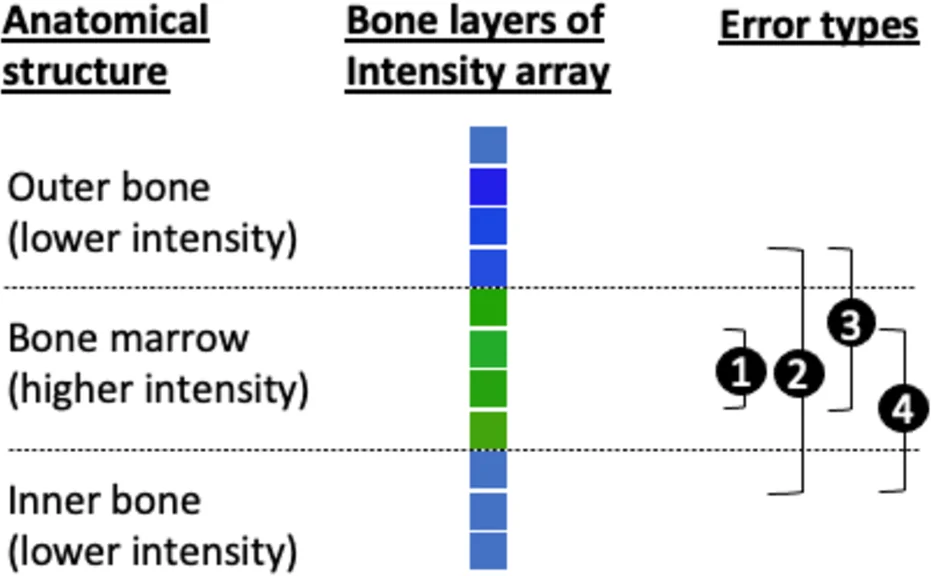
Types of errors in BM localization for a datapoint. The bone marrow is typically more intense than the neighbouring bone, so only errors of type 1 usually result in overestimates of BM intensity for the given datapoint. All other error types (2, 3, and 4) typically result in an underestimate of BM intensity as they incorporate less intense bone layers. This explains why the BM intensity ratio (predicted/true) averaged over all datapoints of a calvarium is centred on 0.95 and not 1 (see also S3).

**Appendix 1—figure 3.**
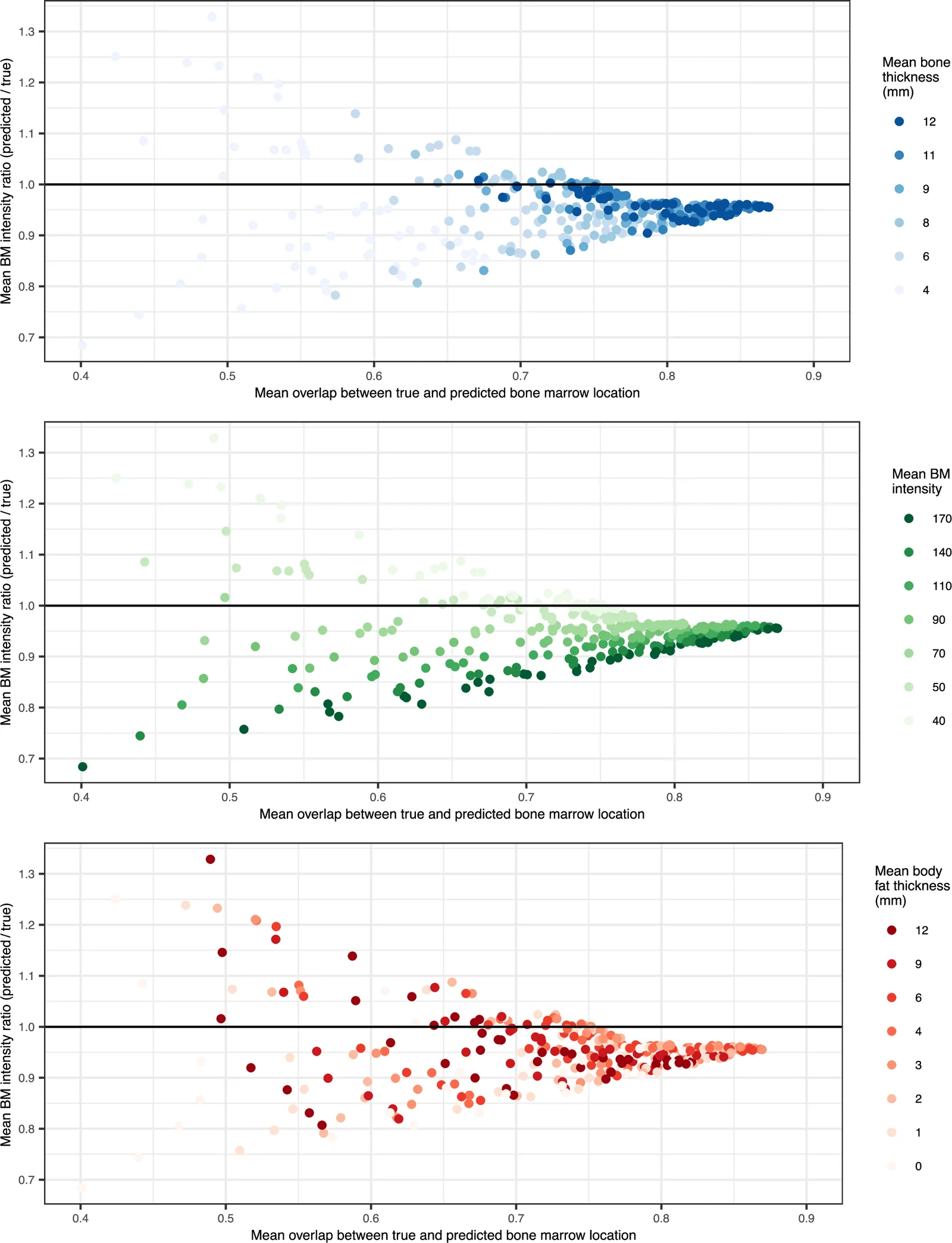
Model performance on 336 simulated head types (6 bone thicknesses, 7 BMA intensities, 8 body fat thicknesses). Top panel: heads coloured by mean bone thickness. Middle panel: heads coloured by BM intensities. Bottom panel: heads coloured by body fat thickness.

**Appendix 1—figure 4.**
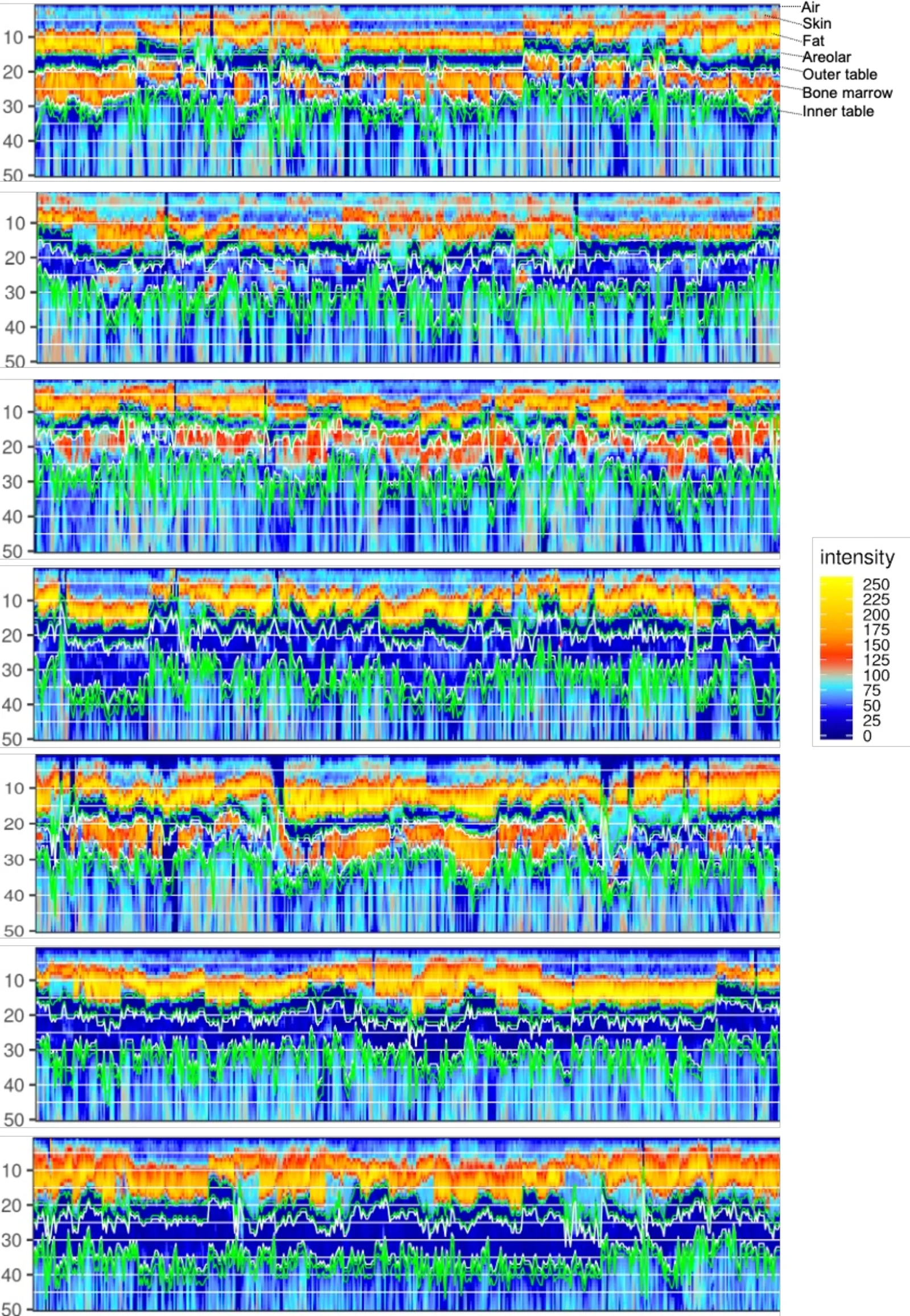
Examples of neural network performance on seven different UK Biobank scans with different levels of subcutaneous fat thickness, bone marrow adiposity, and bone thickness. Outer table (OT), bone marrow (BM), inner table (IT). Predicted boundaries of BM (white lines) and of outer and inner tables (green lines). Adjacent datapoints (*x*-axis) in this plot are not necessarily adjacent on the calvarium.

**Appendix 1—figure 5.**
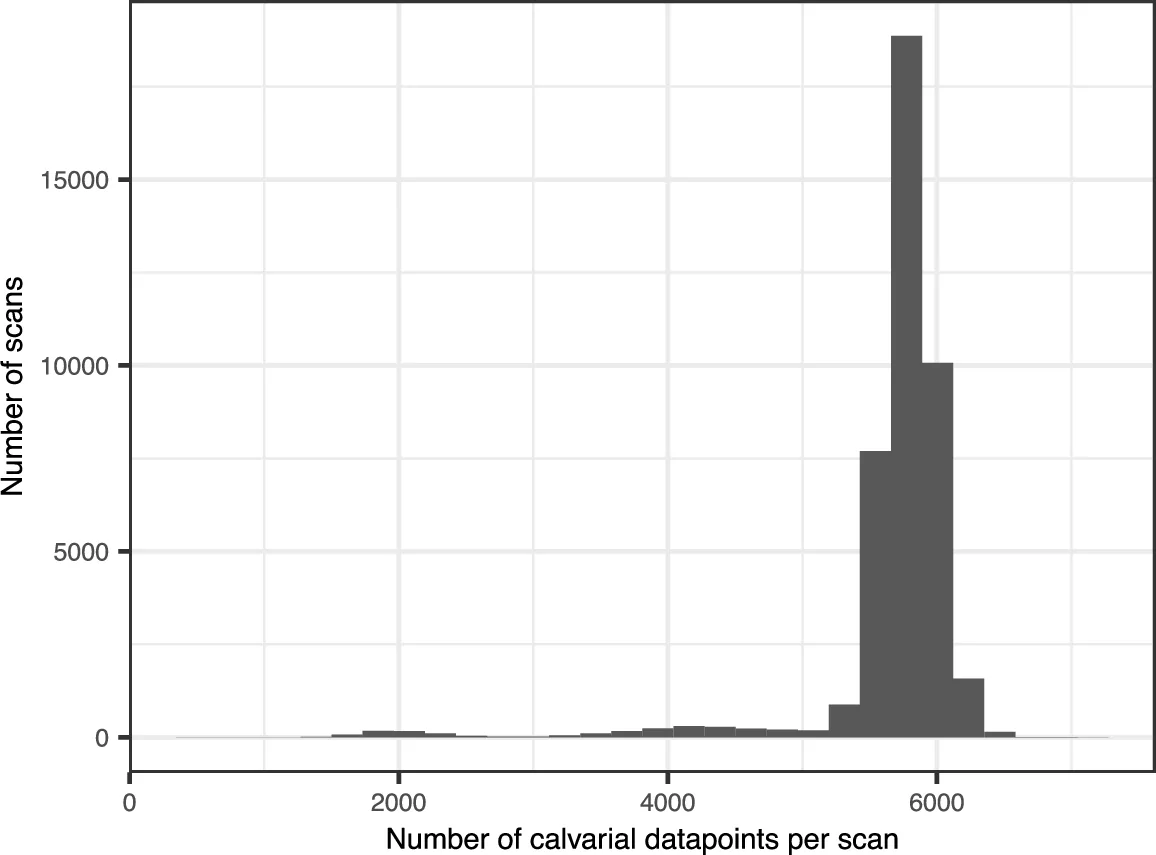
Distribution of number of datapoints per scan (for 41,648 scans). Scans with fewer than 5000 datapoints are likely affected by poor image quality and cannot be used to reliably measure bone marrow intensity.

**Appendix 1—figure 6.**
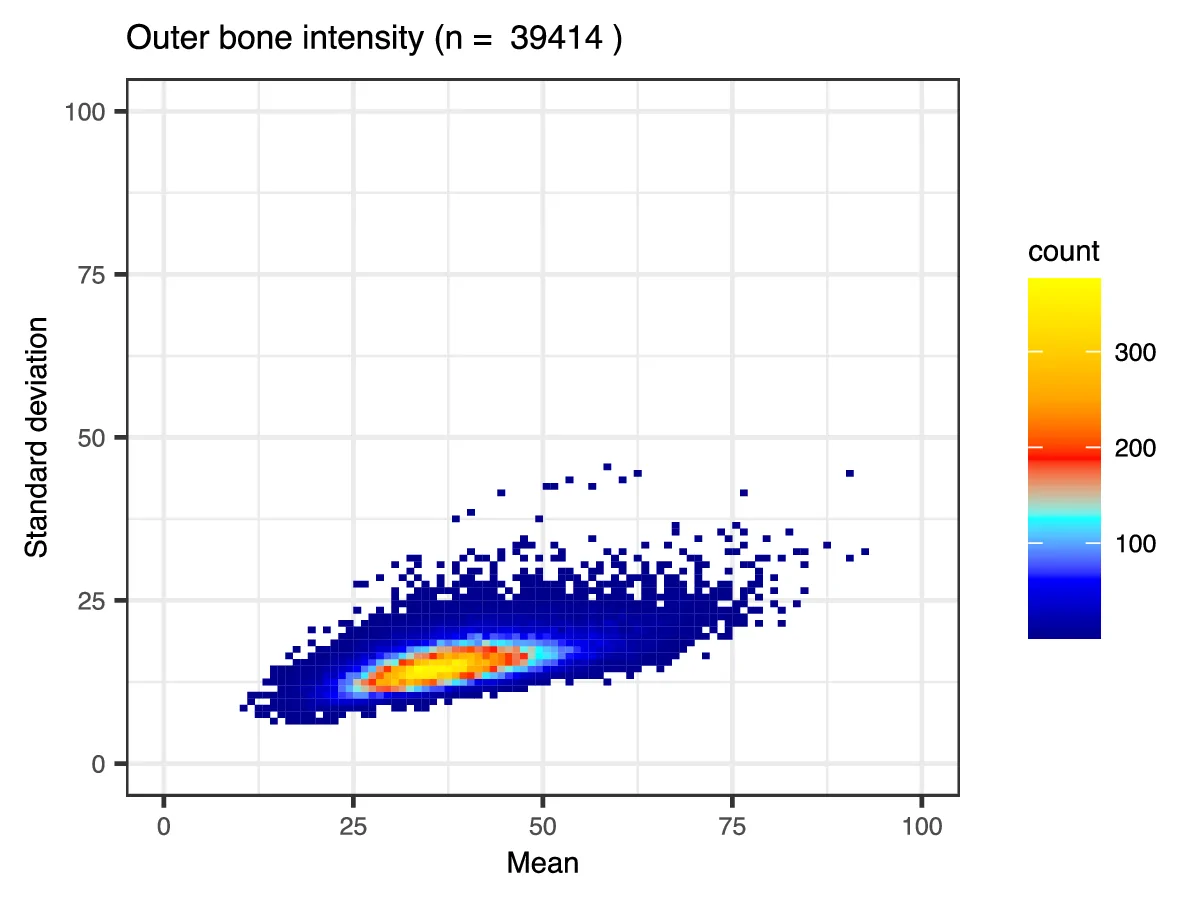
Outer bone intensity mean and standard deviation for 39,414 scans.

**Appendix 1—figure 7.**
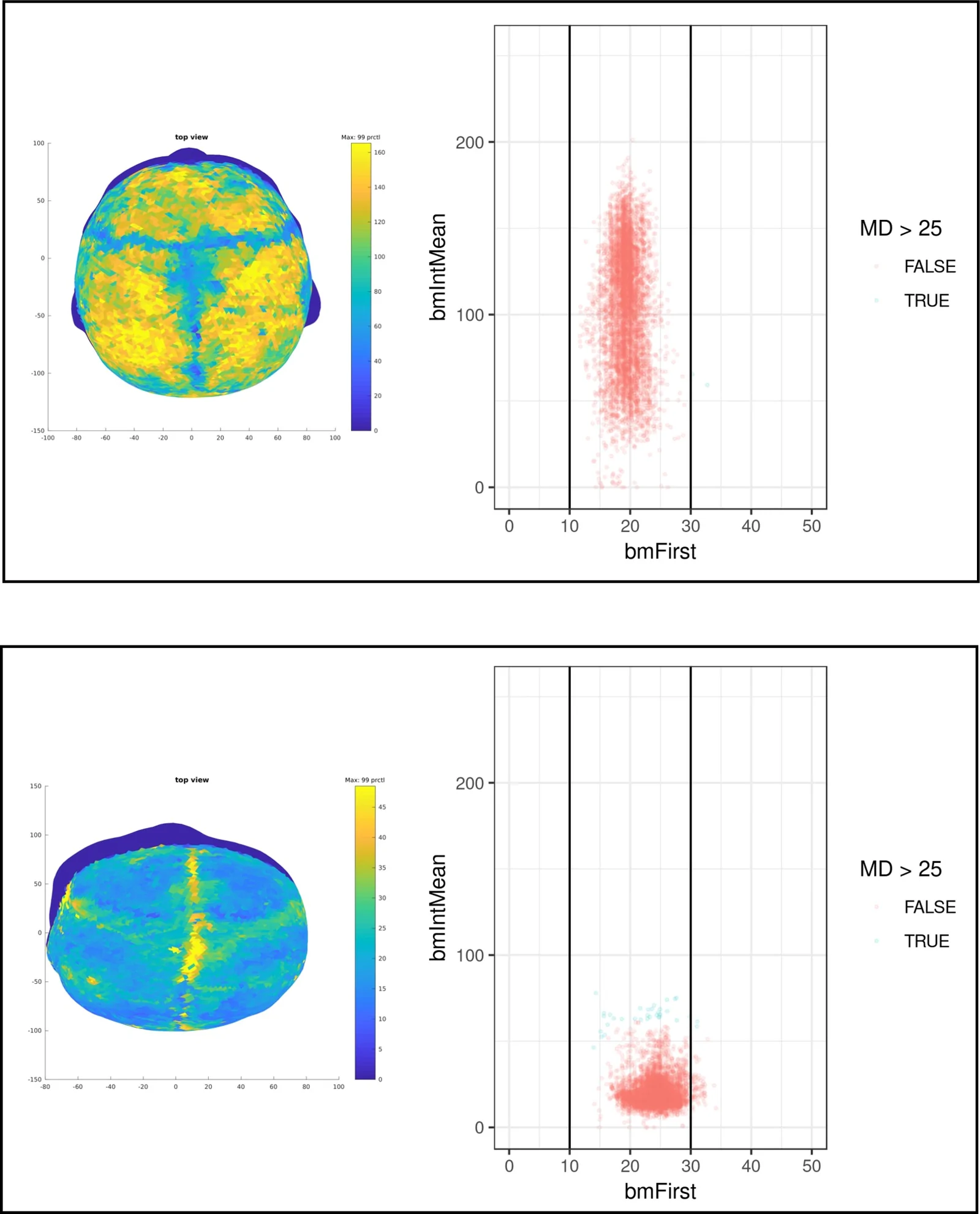
BMA map of the calvarium. BMA map of the calvarium with nose pointing north (left) and first layer of bone marrow plotted against bone marrow intensity for all datapoints in the same scan (right). Top figure for a calvarium with high levels of adiposity, but where no datapoints have a Mahalanobis distance greater than 25. Bottom figure for a calvarium with relatively low levels of adiposity, where the vast majority of points do have a Mahalanobis distance lower than 25 (light red) but where some datapoints have a Mahalanobis distance greater than 25 (light blue). In the corresponding BMA map one can observe that these datapoints with higher intensity are all located on the sagittal suture (which is not high intensity) and correspond to datapoints in which the NN has failed to correctly localize the bone marrow layers.

**Appendix 1—figure 8.**
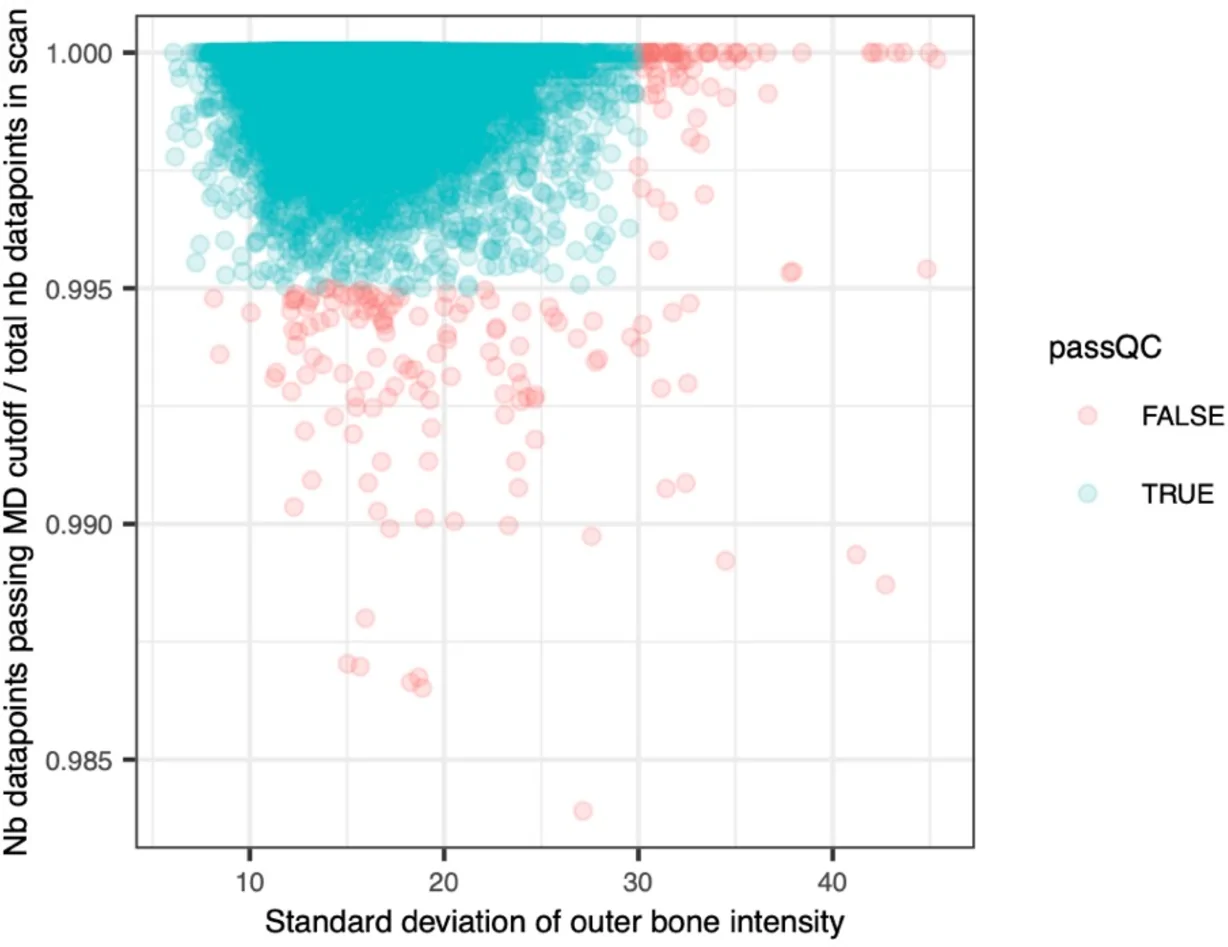
Additional quality control filters. A combination of a maximal value for the standard deviation of outer bone intensity and of a minimum value for the fraction of datapoints passing the Mahalanobis distance cutoff (see previous figure) enables additional QC of correct bone marrow localization.

**Appendix 1—figure 9.**
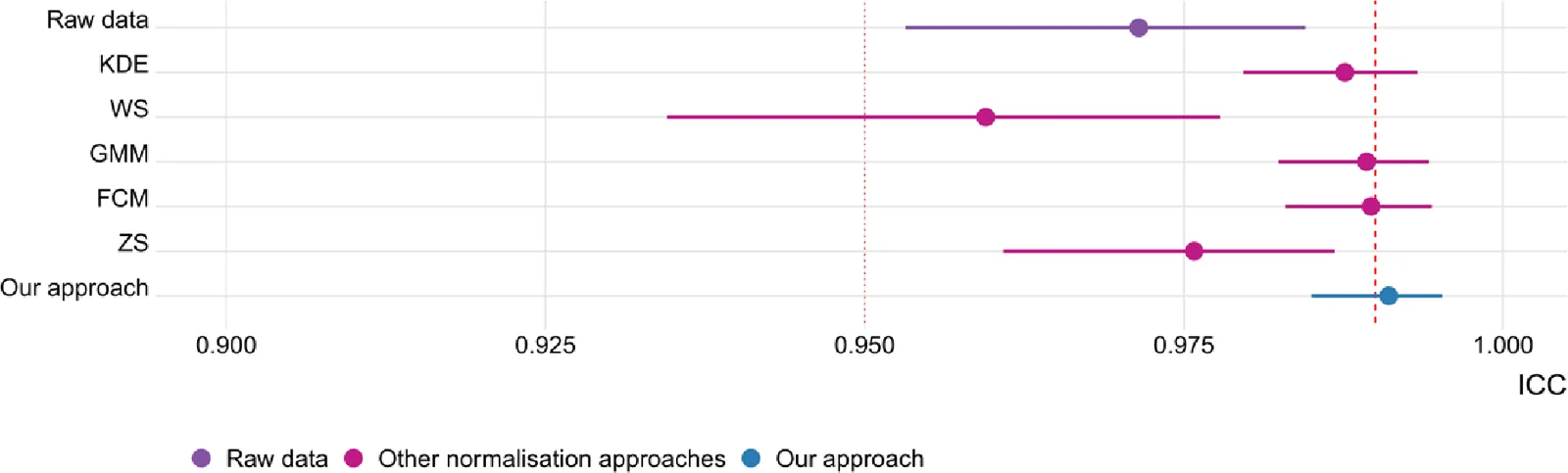
Impact of intensity normalization procedure on test–retest validity. In our proposed pipeline, T1 image intensities were rescaled to assume values between 0 and 255 as part of the recon-all processing stream of FreeSurfer. We compared this normalization approach to five additional normalization procedures that have been extensively used in the image processing literature. Specifically, we performed normalization with Kernel Density Estimation (KDE), WhiteStripe (WS), Gaussian Mixture Model (GMM), Fuzzy *C*-Means (FCM), and *Z*-score normalization (ZS). Details on these methods and their implementation are given in Reinhold et al., 2019. Using test–retest data from the HNU sample, we derived estimates of BMA for each subject and scan (30 individuals, 10 repeat scans) based on each of the five additional normalization approaches. The comparison between normalization strategies shows that several of the normalization strategies yield strong test–retest reliability, yet our approach is on par with, or better than, the other normalization strategies.

**Appendix 1—figure 10.**
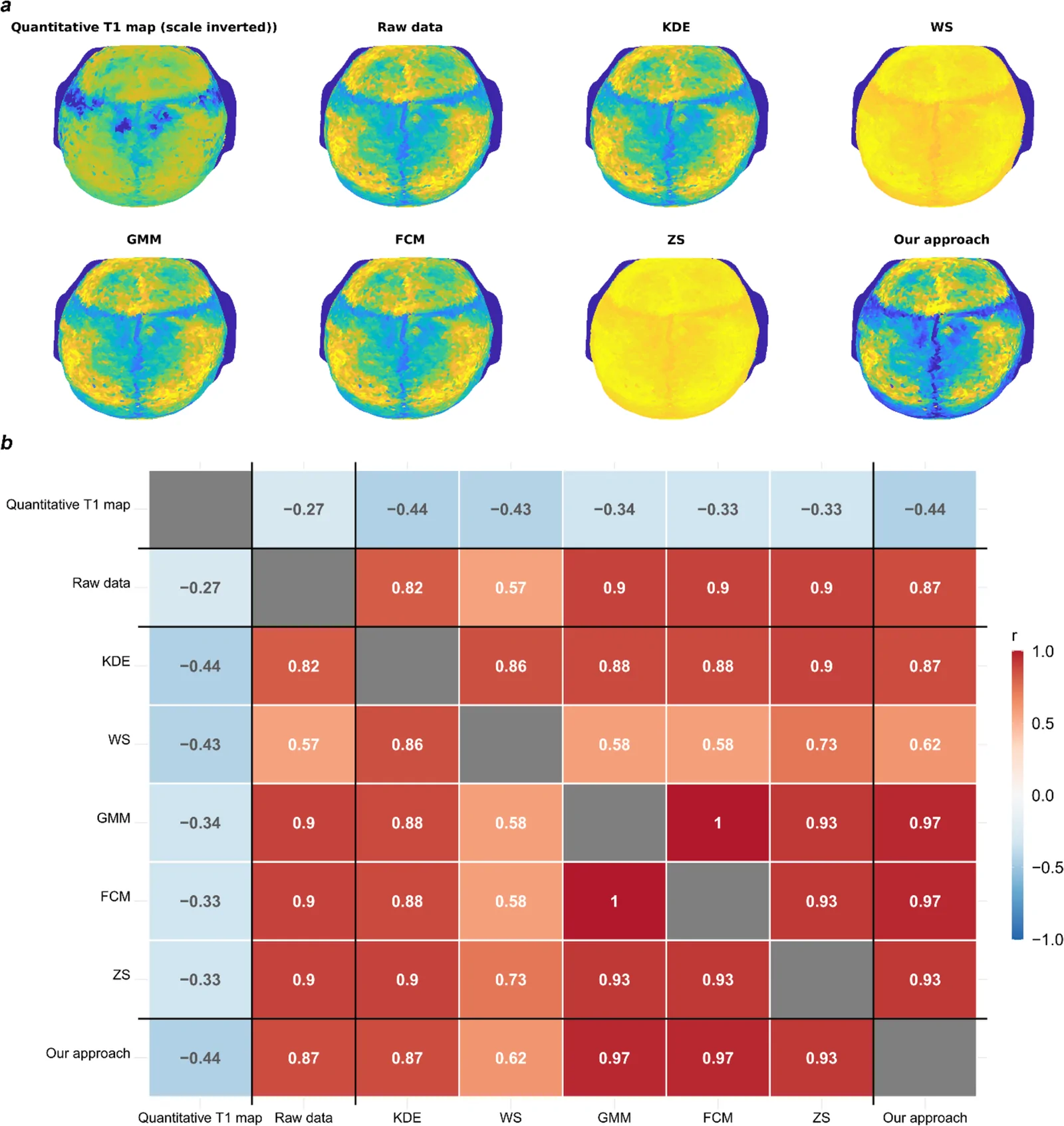
Validation against a quantitative measure of BMA. Data from the MICA-MICs cohort allowed us to compare our BMA estimates to BMA estimates derived from microstructurally sensitive quantitative T1 images acquired with a MP2RAGE MRI sequence. We derived quantitative estimates of BMA from the quantitative T1 images, as well as semi-quantitative estimates based on T1-weighted images after FreeSurfer normalization (our approach) and five additional normalization approaches (KDE, WS, GMM, FCM, and ZS; see also Appendix 1—figure 9). Panel (**a**) illustrates the resulting calvarial maps for an example subject, and panel (**b**) depicts the statistical results. Our BMA estimation approach shows high correlations with estimates derived from T1-weighted images normalized with KDE, GMM, FCM, and ZS, whereas correlations between WS normalization and the other methods are lower. Most importantly, we find significant correlations between the original BMA estimation approach and fully quantitative BMA estimates. The negative correlation here is expected since T1 relaxation times are inversely correlated with fat. These significant correlations corroborate the semi-quantitative nature of our approach. We note that the correlation between quantitative and semi-quantitative approaches, while significant (p = 0.0017), seems moderate (our approach and KDE normalization performing best with *r* = –0.44). However, it is important to emphasize that the age distribution of the MICA-MICs sample is very narrow (29.54 ± 5.62 years) and therefore there is little age-related variance in the derived BMA estimates. We would therefore expect even higher correlations between semi-quantitative and quantitative approaches in datasets with more variation in bone marrow adiposity, such as data covering a wider age span or life events such as menopause.

**Appendix 1—figure 11.**
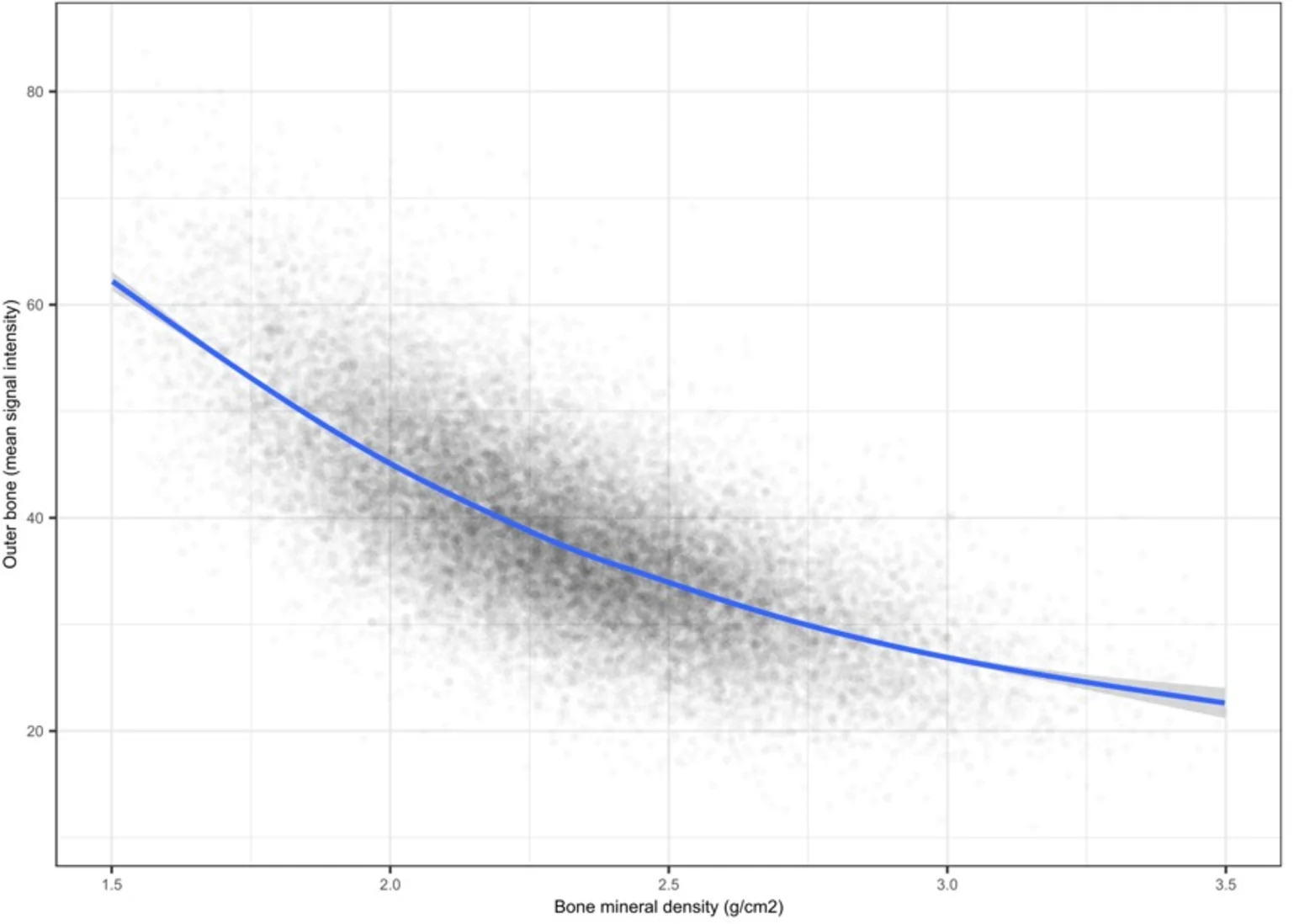
Illustration of the semi-quantitativeness of another measure obtained from T1-weighted MRI. To further illustrate that semi-quantitative approaches from T1-weighted scans can provide plausible measures, we have additionally included an analysis of another calvarial bone measure, specifically the outer-bone intensity. Bone mineral density measures the mineral content of bone in g/cm^2^ and correlates negatively with cortical bone signal intensity in T1-weighted images. We can therefore obtain a second independent estimate of the quantitativeness of signal intensity from the comparison of mean outer-bone signal intensity with calvarial bone mineral density (measured by DEXA scan). We estimate a correlation of –0.715 (CI [-0.72,–0.71]) between these variables in the 29,093 individuals that had both MRI scans and calvarial bone mineral density measurements. This correlation is most likely an accurate estimate of the quantitativeness of the signal intensity as there is a wide distribution of bone mineral density in the 29,093 individuals used for this estimate.

**Appendix 1—figure 12.**
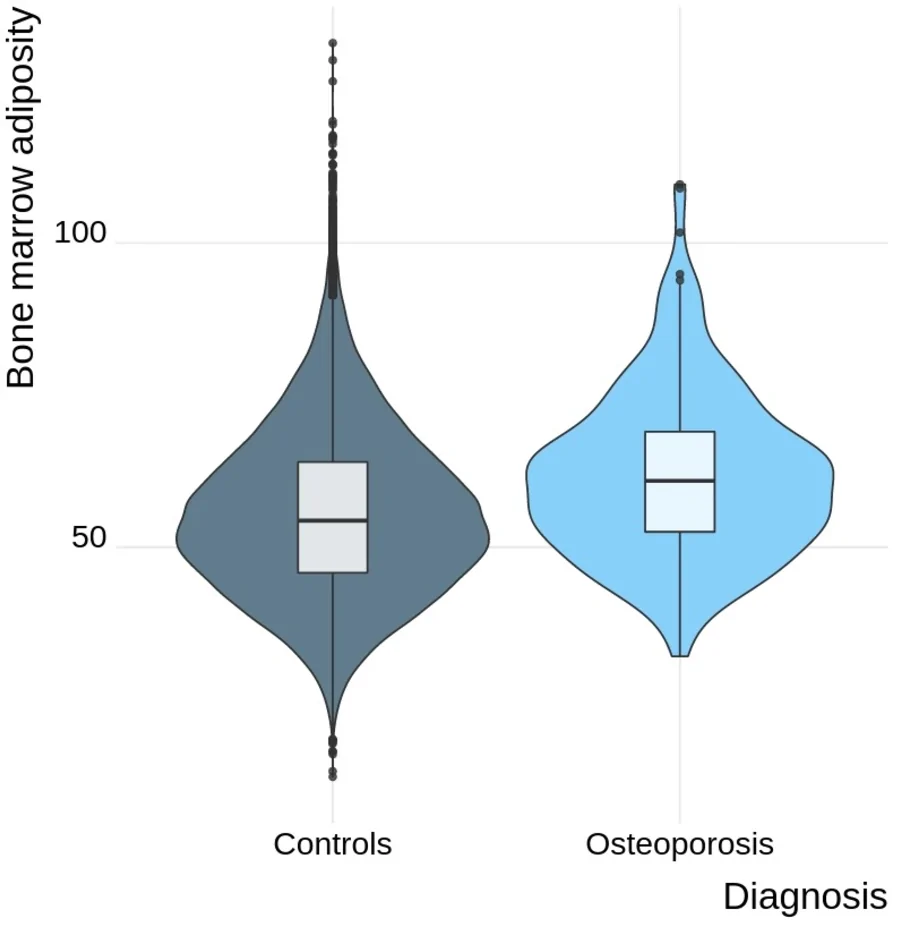
Significant difference in bone marrow adiposity between patients with osteoporosis and control individuals without a diagnosis within the ICD-10 M category. We identified a set of *N* = 220 individuals with an ICD-10 diagnosis of osteoporosis (ICD-10 codes M80-82) in the UK Biobank dataset and compared this group with N=30,127 controls, excluding individuals with any other diagnosis within the M category. In the models adjusted for age, sex, and scanner, BMA was significantly higher in the osteoporosis group (*t* = 9.3; p < 2e−16). Among females, the effect was similar also when controlling for HRT use (*t* = 7.3; p = 3e−13). This analysis shows that our proposed BMA measure allows for robust comparison between subjects and provides another replication of a well-established biological relationship, that is, BMA increase in osteoporosis.

**Appendix 1—figure 13.**
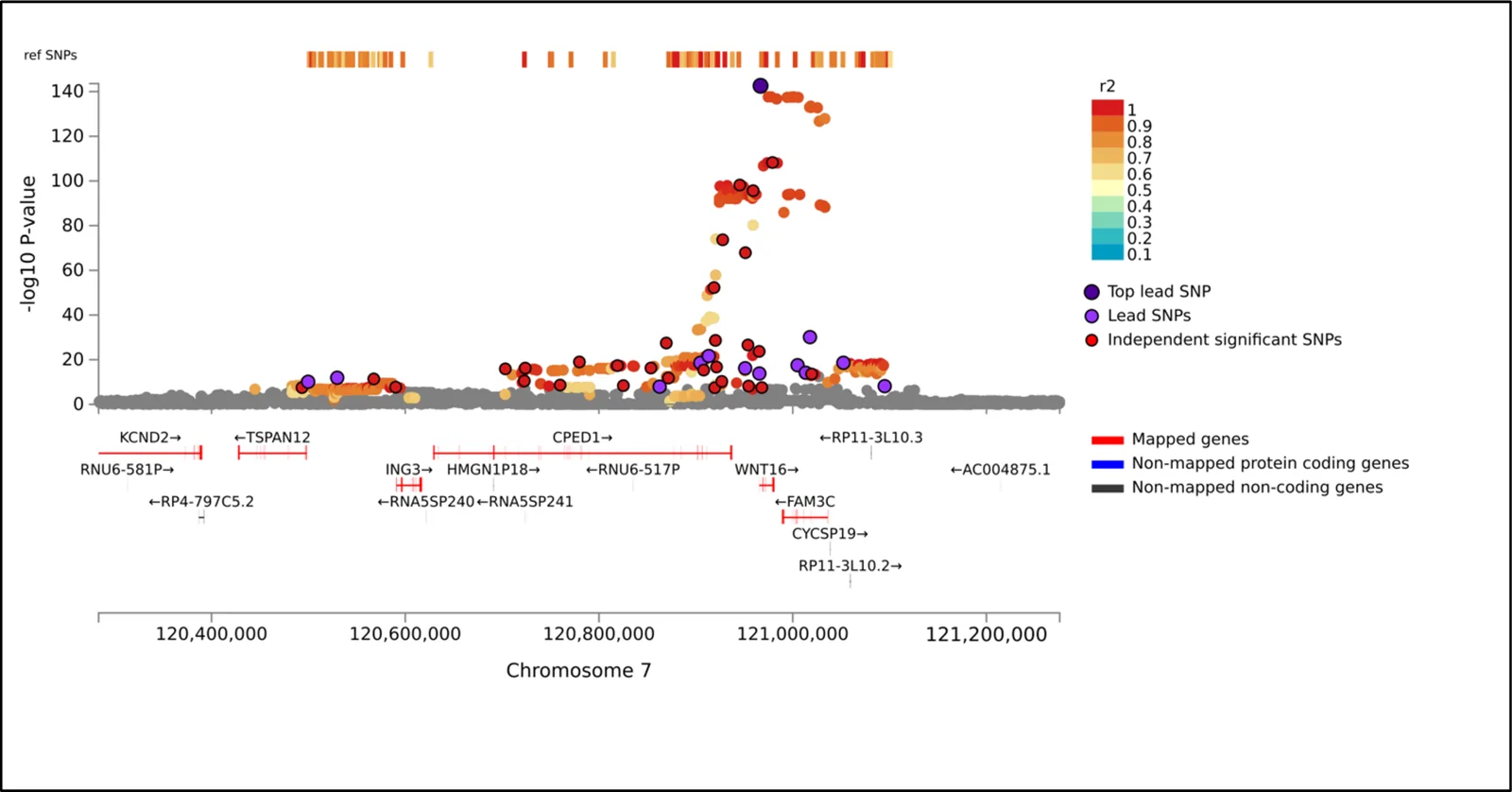
The WNT16 locus in the joint discovery sample.

**Appendix 1—figure 14.**
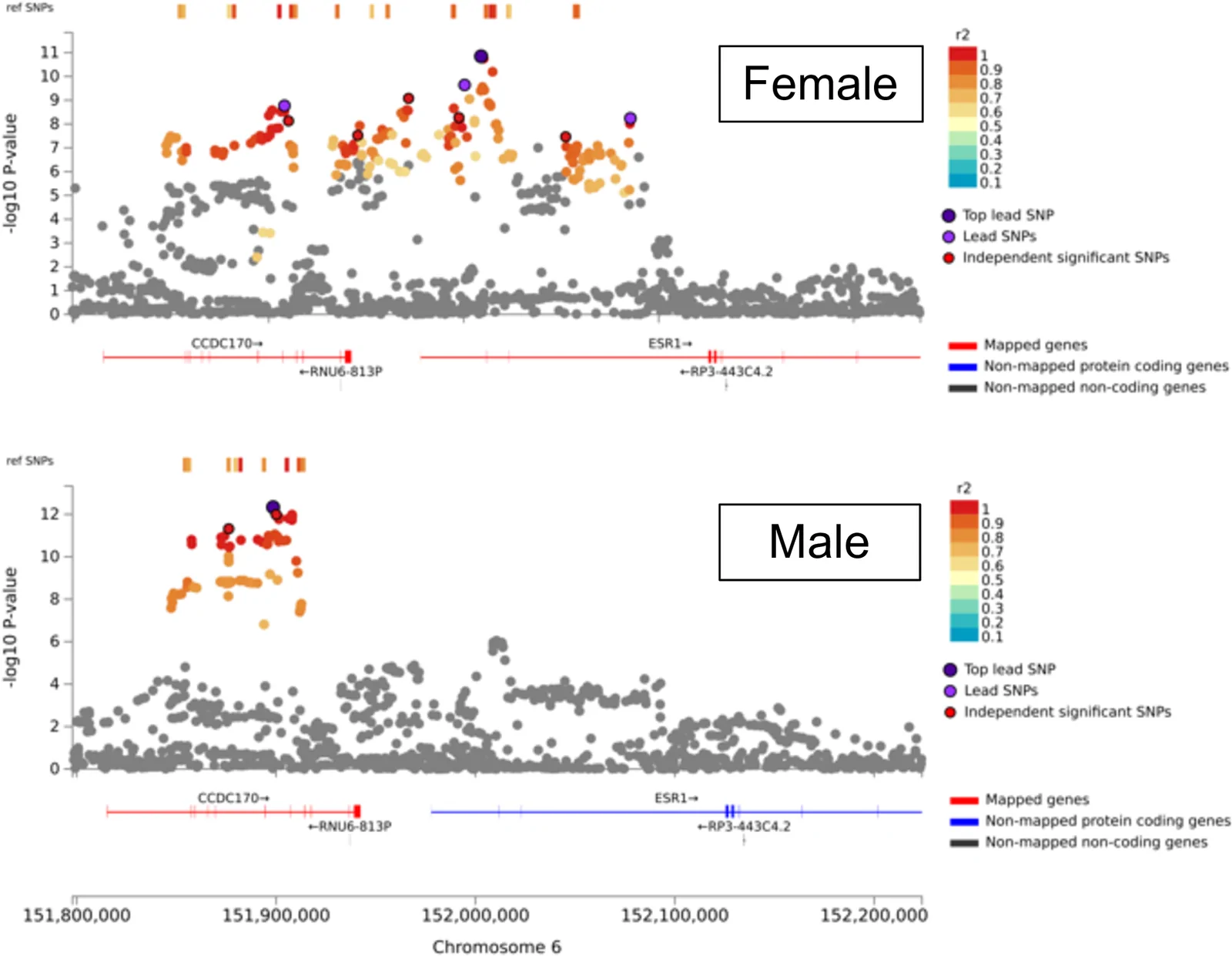
The ESR1 locus in the male and female discovery samples.

## Data Availability

This article only used pre-existing datasets, all of which can be accessed following an approved application. All data are available in the main text or the supplementary materials. This research has been conducted using the UK Biobank Resource (Bycroft et al., 2018) (access code 27412), the Human Connectome Project (Van Essen et al., 2013), data from Hangzhou Normal University as part of the data provided by the Consortium for Reliability and Reproducibility (Zuo et al., 2014), and the MICA-MICs dataset, which is available on the Canadian Open Neuroscience Platform's data portal. Human Connectome Project data was provided by the WU-Minn Consortium (Principal Investigators: David Van Essen and Kamil Ugurbil; 1U54MH091657) funded by the 16 NIH Institutes and Centers that support the NIH Blueprint for Neuroscience Research;, and by the McDonnell Center for Systems Neuroscience at Washington University. We obtained GWAS summary statistics from the following consortia: Genetic Factors for Osteoporosis Consortium, Genetic Investigation of ANthropometric Traits, ICBP International Consortium for Blood Pressure, Diabetes Genetics Replication Andand Meta- analysis Consortium, Coronary Artery Disease Genome-wide Replication and Meta-analysis Consortium, Complex Traits Genetics Consortium, Social Science Genetic Association Consortium, International Multiple Sclerosis Genetics Consortium, International Parkinson Disease Genomics Consortium, Psychiatric Genomics Consortium. Summaries of BMA GWAS statistics are available on the FUMA website (male+female: ID 153909, male: ID 153916, female: ID 153914) and complete GWAS statistics are available from https://github.com/comgen/boneMarrowLocalisationT1wMRI, copy archived at Hughes, 2024. The following previously published datasets were used: RoyerJ Rodriguez-CrucesR TavakolS LariviereS HerholzP LiQ Vos de WaelR PaquolaC BenkarimO ParkB LoweAJ MarguliesDS SmallwoodJ BernasconiA BernasconiN FrauscherB BernhardtBC 2022MICA-MICs: a dataset for Microstructure-Informed ConnectomicsCONP Portalmica-mics ZhongL YaoL TowerRJ WeiY MiaoZ ParkJ ShresthaR WangL YuW HoldreithN ZhangY TongW GongY AhnJ SusztakK DymentN LiM LongF SealeP QinL 2020Single cell transcriptomics identifies a unique adipose lineage cell population that regulates the bone marrow environmentSingle Cell PortalSCP101710.7554/eLife.54695PMC722038032286228

## Additional files

MDAR checklist

## Appendix 1

### Tool workflow for calvarial bone marrow detection

Main steps:

1. **Identify skin surface** using watershed https://surfer.nmr.mgh.harvard.edu/fswiki/mri_watershed.
2. **Identify calvarium** based on brain topology: compute mask in Matlab by reading in the parcellation from freesurfer (‘aseg’ file) and identifying the relevant region borders for masking the image.
3. **Extract intensity array** for every calvarium datapoint: intensity values are extracted from nu.mgz in a stepwise run from the outer skin surface inwards using mri_vol2surf from Freesurfer.
4. **Localize bone marrow** for each datapoint by applying the artificial neural network model to the datapoint’s intensity array (Run_predBM in python).
5. **Average intensities located in bone marrow** for every datapoint (computeAuxiliaryFiles in R).
6. **Plot average BMA on calvarial skin surface** using Matlab (own code which draws the BMA values on the surface mesh of the outer skin surface).
7. **Average BM intensities** over all calvarium datapoints.

Software versions:

- Matlab: 2018a
- Freesurfer: 5.3.0
- R: 3.5.0
- Python: Anaconda3/5.3.0, python 3.8.1, tensorflow 2.4.1

### Twin-based heritability estimates in HCP

**Appendix 1—table 1.** Twin-based heritability estimates using the NACE model fitted in R using the twinlm() function of the mets package. NACE: normal (N) additive genetic effects (A), common environment (C), the unique environment (E). Female pairs aged 26–36 and male pairs aged 22–34. The Falconer method produced similar heritability estimates.

NACE	A	C	E
Males (114 MZ and 60 DZ twins)	0.91	0.00	0.09
Females (160 MZ and 96 DZ twins)	0.60	0.22	0.18

### BMA mediation analysis

**Appendix 1—table 2.** OLS regression analysis of the determinants of BMD (in males and females). Two models are shown for females, one including BMA (left) and one excluding BMA (right). Age and HRT have statistically significant effects on BMD when BMA is excluded. The coefficient on HRT drops by a factor of 20 and loses statistical significance when BMA is included in the regression, and *R*^2^ of the regression increases by a factor of 4, thus indicating that the effect of HRT on BMD is largely mediated by HRT’s effect on BMA. The number of samples is somewhat reduced because BMD data was not available for all samples with BMA data.

BMD explanatory variables	Males		Females		Females	
	**Coeff**.	**p-value**	**Coeff**.	**p-value**	**Coeff**.	**p-value**
Intercept	2.873	<2e−16	3.161	<2e−16	3.028	<2e−16
BMA	–0.006	<2e−16	–0.012	<2e−16	Excluded	
Age	–0.002	5.66E−15	–0.004	<2e−16	–0.012	<2e−16
Scanner_1	0.086	<2e−16	0.063	<2e−16	0.048	1.07E−08
Scanner_2	0.016	0.00756	0.003	0.612	–0.005	0.464
EverHRT	Not relevant		0.002	0.75	0.034	1.16E−09
*R*-squared	0.094	<2e−16	0.303	<2e−16	0.071	<2e−16
*N*	14,262		14,831		14,831	

### GWAS quality control and genome-wide significant loci

**Appendix 1—table 3.** Characteristics of the discovery and replication samples.

Discovery is white British							
Replication is cross-ethnic							
**Age**	**Min**	**1st Q**	**Median**	**Mean**	**3rd Q**	**Max**	**Standard deviation**
Discovery	45.13	58.51	64.94	64.33	70.24	82.06	7.51
Replication	44.57	56.69	62.93	62.89	68.87	81.07	7.706
**Sex**	**Male**	**Female HRT**	**Female no HRT**	**Total**			
Discovery	16,140	6373	10,529	33,042			
Replication	2338	874	1746	4958			
**Scanner**	**UKBB_11027**	**UKBB_11025**	**UKBB_11026**	**Total**			
Discovery	8514	20,652	3876	33,042			
Replication	794	2898	1266	4958			

**Appendix 1—table 4.** LDSR estimates of *h*^2^_SNP_ and genomic inflation.

	Discovery GWASes						Replication GWAS	
	Male		Female		Both sexes		Both sexes	
	Statistic	Error	Statistic	Error	Statistic	Error	Statistic	Error
**Total observed scale *h*^2^**	0.4259	0.0519	0.2326	0.0376	0.3148	0.0329	0.2859	0.1002
**Lambda GC**	1.1082		1.0833		1.1587		0.9809	
**Mean chi^2^**	1.1407		1.1148		1.2457		0.989	
**Intercept**	1.0031	0.0085	1.037	0.0075	1.0382	0.0093	0.9602	0.006
**Ratio**	0.0223	0.0602	0.3225	0.0657	0.1554	0.0379	NA	(mean chi^2^ < 1)

**Appendix 1—table 5.** Discovery (both sexes) genome–wide significant loci and lead SNP for each locus (third column), with effect size, standard error, and p-value for these SNPs in the male only, female only, and replication (non-white ethnicity, both sexes) samples. In grey, lead SNPs that are GWS in males but not in females. In pink, lead SNPs with opposite effect sign in the discovery (male + female) vs the cross-ethnic replication (male + female). 92.7% of the lead SNPs of the discovery sample showed the same effect direction in the replication sample.

							Female			Male			Male and female			Replication		
**Locus ID**	**SNP**	**CHR**	**BP**	**A1**	**A2**	**Nearest gene**	**BETA**	**SE**	**p-value**	**BETA**	**SE**	**p-value**	**BETA**	**SE**	**p-value**	**BETA**	**SE**	**p-value**
1	rs6679981	1	22697860	A	G	WNT4	–1.37	0.18	1.79E−14	–1.35	0.19	1.95E–12	–1.36	0.13	2.76E–25	–0.53	0.33	1.10E–01
2	rs6760396	2	71954468	A	G	DYSF	0.66	0.15	5.44E–06	0.85	0.16	4.49E–08	0.75	0.11	1.27E–12	–0.07	0.28	8.03E–01
3	rs11675489	2	85498783	G	A	TCF7L1	0.8	0.14	9.15E–09	0.71	0.15	1.98E–06	0.75	0.1	1.21E–13	0.24	0.27	3.70E–01
4	rs10188985	2	85941074	A	G	GNLY	–0.46	0.14	1.05E–03	–0.69	0.15	4.81E–06	–0.57	0.1	2.72E–08	–0.2	0.27	4.58E–01
5	rs13427880	2	101783867	C	T	TBC1D8	–0.74	0.15	6.84E–07	–0.47	0.16	3.46E–03	–0.61	0.11	2.75E–08	–0.54	0.29	6.23E–02
6	rs13415101	2	166564362	C	T	CSRNP3	0.53	0.14	1.03E–04	0.61	0.15	3.45E–05	0.57	0.1	1.37E–08	0.07	0.26	8.06E–01
7	rs12472217	2	242126109	C	T	ANO7	0.5	0.14	4.55E–04	0.69	0.15	7.52E–06	0.59	0.1	1.59E–08	0.53	0.28	5.76E–02
8	rs4505759	4	1003022	T	C	FGFRL1	–0.72	0.15	1.35E–06	–0.96	0.16	1.75E–09	–0.84	0.11	1.55E–14	–0.59	0.29	4.55E–02
9	rs1833517	5	55784364	G	A	CTC-236F12.4	1.07	0.25	1.97E–05	1.01	0.27	1.42E–04	1.04	0.18	1.18E–08	1.57	0.43	2.68E–04
10	rs12211905	6	74457833	G	T	CD109	–0.31	0.14	2.67E–02	–0.97	0.15	1.18E–10	–0.63	0.1	7.57E–10	–0.52	0.27	5.46E–02
11	rs13194508	6	127144683	C	T	CENPW	0.58	0.16	1.91E–04	0.94	0.17	2.08E–08	0.76	0.11	3.62E–11	0.98	0.33	3.01E–03
12	rs4870045	6	151908047	A	G	CCDC170	0.91	0.15	1.70E–09	1.16	0.16	1.22E–12	1.03	0.11	1.53E–20	0.49	0.27	6.84E–02
13	rs10272752	7	38118313	T	C	SFRP4	–0.87	0.15	2.17E–08	–1.16	0.16	1.65E–12	–1.01	0.11	2.94E–19	–1.36	0.3	5.66E–06
14	rs12704872	7	96334616	C	T	SHFM1	0.46	0.14	9.07E–04	0.66	0.15	9.04E–06	0.56	0.1	3.93E–08	0.38	0.27	1.50E–01
15	rs45446698	7	99332948	G	T	CYP3A7	1.96	0.33	3.50E–09	1.24	0.36	6.46E–04	1.61	0.25	4.93E–11	1.92	0.77	1.33E–02
16	rs3779381	7	120966790	G	A	WNT16	–2.86	0.15	2.08E–75	–2.98	0.17	5.43E–70	–2.92	0.11	2.61E–143	–2.59	0.3	4.28E–18
17	rs330060	8	9090902	G	A	PPP1R3B	0.42	0.14	2.18E–03	0.8	0.15	5.21E–08	0.61	0.1	1.55E–09	0.61	0.26	2.01E–02
18	rs17744726	8	11411823	A	G	BLK	0.42	0.15	6.10E–03	0.97	0.16	2.62E–09	0.69	0.11	6.19E–10	0.27	0.29	3.54E–01
19	rs73229843	8	22501409	C	G	BIN3	–1.53	0.41	1.63E–04	–2.5	0.44	1.56E–08	-2	0.3	2.49E–11	–1.92	0.86	2.64E–02
20	rs1283688	8	108416308	C	T	ANGPT1	0.42	0.14	3.00E–03	0.77	0.15	3.13E–07	0.59	0.1	9.58E–09	0.27	0.27	3.16E–01
21	rs11995824	8	120012700	C	G	COLEC10	–0.9	0.14	7.84E–11	–0.56	0.15	1.79E–04	–0.73	0.1	5.30E–13	–1.06	0.27	7.92E–05
22	rs117474778	9	24200738	T	C	IZUMO3	1.57	0.39	4.80E–05	1.64	0.43	1.13E–04	1.61	0.29	2.13E–08	–1.15	0.78	1.43E–01
23	rs514659	9	136142203	C	A	OBP2B	0.77	0.15	1.78E–07	0.65	0.16	4.18E–05	0.71	0.11	4.86E–11	0.39	0.28	1.56E–01
24	rs4994332	9	137117194	T	C	RXRA	0.83	0.14	4.56E–09	0.44	0.15	3.64E–03	0.64	0.1	6.65E–10	0.74	0.27	7.01E–03
25	rs10826409	10	28449221	T	G	MPP7	–0.81	0.14	1.98E–08	–0.67	0.15	1.03E–05	–0.74	0.1	1.53E–12	–0.09	0.26	7.18E–01
26	rs17476364	10	71094504	C	T	HK1	–0.46	0.22	3.77E–02	–1.42	0.24	1.97E–09	–0.93	0.16	8.41E–09	–0.24	0.49	6.24E–01
27	rs1463597	10	81139141	G	A	ZCCHC24	–0.44	0.14	1.37E–03	–0.73	0.15	1.10E–06	–0.58	0.1	1.06E–08	–0.73	0.27	6.26E–03
28	rs7945211	11	27472777	T	C	LGR4	–0.64	0.14	3.72E–06	–0.87	0.15	5.84E–09	–0.75	0.1	1.39E–13	–0.42	0.26	1.06E–01
29	rs10769239	11	47130540	G	C	C11orf49	–0.54	0.15	2.24E–04	–0.77	0.16	1.08E–06	–0.65	0.11	1.32E–09	–0.21	0.28	4.51E–01
30	rs1352075	11	69457293	T	C	CCND1	–0.41	0.14	2.76E–03	–0.84	0.15	1.54E–08	–0.62	0.1	7.96E–10	–0.12	0.26	6.58E–01
31	rs61909068	11	128342957	G	A	ETS1	0.54	0.19	3.64E–03	1.02	0.2	5.58E–07	0.77	0.14	2.13E–08	0	0.39	9.95E–01
32	rs12311504	12	28478771	G	A	CCDC91	0.49	0.14	4.46E–04	1.22	0.15	6.98E–16	0.85	0.1	1.87E–16	1.21	0.27	8.43E–06
33	rs10875906	12	49385679	T	C	DDN	–0.56	0.16	3.74E–04	–0.79	0.17	4.17E–06	–0.67	0.12	7.45E–09	–0.86	0.3	4.51E–03
34	rs138818878	13	43148546	G	C	TNFSF11	–2.15	0.43	6.33E–07	–1.64	0.46	4.17E–04	–1.9	0.32	1.91E–09	1.02	0.99	3.03E–01
35	rs9543735	13	75213211	A	C	AL355390.1	–0.63	0.14	1.32E–05	–0.55	0.16	4.05E–04	–0.59	0.11	2.48E–08	–0.5	0.28	7.32E–02
36	rs762643	14	54422767	T	G	BMP4	0.71	0.14	1.93E–07	0.46	0.15	2.07E–03	0.59	0.1	5.28E–09	0.18	0.27	5.06E–01
37	rs2414098	15	51537806	T	C	CYP19A1	0.3	0.14	3.27E–02	0.92	0.15	1.94E–09	0.61	0.1	7.01E–09	0.46	0.28	9.99E–02
38	rs11870439	17	833790	T	G	NXN	–0.94	0.14	4.78E–11	–1.16	0.15	3.28E–14	–1.05	0.1	1.19E–23	–0.9	0.28	1.39E–03
39	rs112698747	17	47948829	G	A	TAC4	–2.01	0.31	8.43E–11	–0.92	0.35	7.57E–03	–1.5	0.23	9.05E–11	–2.05	0.71	3.77E–03
40	rs3790161	20	10640090	T	A	JAG1(SNP overlaps JAG1)	0.95	0.14	4.63E–12	0.88	0.15	2.19E–09	0.92	0.1	8.12E–20	1.05	0.26	7.52E–05
41	rs34952318	20	11177055	A	G	JAG1 (SNP 536 kbp from JAG1)	1.58	0.32	6.10E–07	1.84	0.34	5.47E–08	1.71	0.23	1.63E–13	0.84	0.66	2.07E–01

­

**Appendix 1—table 6.** Comparison of p-values between the ’both sexes’ discovery sample and the male, female, and replication (both sexes) samples.

	p < 5e−8			p < 5e−2		
	Male	Female	Replication	Male	Female	Replication
Based on all discovery SNPs that are p < 5e−8	42.0%	31.3%	3.1%	100.0%	99.5%	62.2%
Based on one lead SNP for each of the 41 loci	41.5%	29.3%	2.4%	100.0%	100.0%	39.0%

### Single-cell RNAseq data from mouse bone marrow

**Appendix 1—table 7.** Human genes for which expression data was located in the Zhong dataset. Closest indicates that the gene is closest to the lead SNP in a locus. Genes in the table not marked closest are genes for which SNPs in the locus are strong eQTLs.

Human gene	Mouse gene	Closest
ANGPT1	Angpt1	Closest
BIN3	Bin3	Closest
BLK	Blk	Closest
BMP4	Bmp4	Closest
CCDC170	Ccdc170	Closest
CCDC91	Ccdc91	Closest
CCND1	Ccnd1	Closest
CD109	Cd109	Closest
CENPW	Cenpw	Closest
CPED1	Cped1	
CSRNP3	Csrnp3	Closest
CYP26B1	Cyp26b1	
DLX5	Dlx5	
DLX6	Dlx6	
DYSF	Dysf	Closest
ESR1	Esr1	
ETS1	Ets1	Closest
FAM167A	Fam167a	
FAM3C	Fam3c	
FGFRL1	Fgfrl1	Closest
GALNT3	Galnt3	
HK1	Hk1	Closest
JAG1	Jag1	Closest
LGR4	Lgr4	Closest
LRP4	Lrp4	
MPP7	Mpp7	Closest
NXN	Nxn	Closest
ORAOV1	Oraov1	
PASK	Pask	
PPIF	Ppif	
PPP1R3B	Ppp1r3b	Closest
RXRA	Rxra	Closest
SFRP4	Sfrp4	Closest
SHFM1	Shfm1	Closest
SURF1	Surf1	
TBC1D8	Tbc1d8	Closest
TCF7L1	Tcf7l1	Closest
TNFRSF11B	Tnfrsf11b	
TNFSF11	Tnfsf11	Closest
WNT16	Wnt16	Closest
WNT4	Wnt4	Closest
ZCCHC24	Zcchc24	Closest

**Appendix 1—table 8.** Human genes for which expression data was not located in the Zhong dataset.

Human gene	Mouse gene	Closest	Reason for failure to locate expression in mouse scRNAseq data
ANO7	Ano7	Closest	Gene close to zero expression in the Zhong dataset so it is considered artifact noise.
c11orf49	1110051M20Rik	Closest	No expression detected.
COLEC10	Colec10	Closest	No expression detected. GTEx RNA-seq data show zero expression in bone marrow.
AL355390.1	N/A	Closest	Human-specific uncategorized lncRNA.
CTC-236F12.4	N/A	Closest	Human-specific uncategorized lncRNA.
CYP19A1	Cyp19a1	Closest	No expression detected.
CYP3A7	Cyp3a13	Closest	Gene close to zero expression in the Zhong dataset so it is considered artifact noise.
DDN	Ddn	Closest	Gene close to zero expression in the Zhong dataset so it is considered artifact noise.
GNLY	N/A	Closest	Human, chimpanzee, cow, and dog. No ortholog in mouse.
IZUMO3	Izumo3	Closest	No expression detected.
OBP2B	Obp2a	Closest	No expression detected.
TAC4	Tac4	Closest	No expression detected.

### Genetic overlap between BMA and other traits at the locus level

**Appendix 1—table 9.** Akaike information criterion values for all MiXeR models. Positive best_vs_min_overlap AIC estimates indicate that MiXeR can accurately distinguish the reported overlap from the minimum possible overlap allowed. Positive best_vs_max_overlap AIC estimates indicate that MiXeR can accurately distinguish the reported overlap from the maximum possible overlap allowed.

Trait	Genetic correlation (LDSR)							Genetic overlap (MiXeR)					Akaike information criterion	
	Male		Female		All subjects			Partitioning of causal variants Mean nb per partition (SE)			Overlap as fraction of all BMA causal variants	Corr. of effect sizes within overlap (SE)		
	*r*_g_	p	*r*_g_	p	*r*_g_	p	*Q*	Unique to BMA	Overlap	Unique to trait			Best vs min	Best vs max
Bone mineral density	–0.36	**1.4E–14**	–0.47	**2.6E–17**	–0.41	**1.5E–22**	**2.0E–21**	3 (3)	497 (58)	1471 (56)	**0.99**	**–0.95 (0.05)**	13.36	2.47
Body mass index	–0.18	**3.9E–09**	–0.24	**5.1E–09**	–0.20	**9.3E–15**	**6.1E–14**	NA*	NA*	NA*	NA*	NA*	NA*	NA*
Waist-to-hip ratio	–0.092	5.0E–02	–0.103	6.0E–02	–0.10	1.2E–02	2.60E–02	63 (74)	436 (93)	3300 (466)	**0.87**	–0.48 (0.16)	5.75	1.30
Blood pressure systolic	–0.02	7.0E–01	–0.03	5.5E–01	–0.02	6.5E–01	6.8E–01	25 (22)	475 (63)	3921 (78)	**0.95**	–0.25 (0.05)	45.31	4.85
Blood pressure diastolic	–0.03	4.6E–01	–0.05	2.5E–01	–0.03	3.4E–01	4.0E–01	27 (31)	473 (69)	3541 (90)	**0.95**	–0.21 (0.04)	58.37	2.77
Coronary artery disease	–0.09	7.6E–02	–0.10	1.2E–01	–0.10	3.6E–02	6.6E–02	275 (48)	225 (57)	1140 (95)	0.45	–0.40 (0.11)	8.53	24.01
TypE–2 diabetes	–0.09	3.3E–02	–0.04	4.3E–01	–0.07	6.5E–02	1.1E–01	361 (67)	139 (31)	752 (49)	0.28	**–0.69 (0.16)**	8.75	77.44
Insomnia	–0.04	3.5E–01	–0.05	4.2E–01	–0.05	2.1E–01	2.7E–01	248 (144)	252 (161)	8868 (614)	0.50	–0.45 (0.32)	2.49	3.57
Multiple sclerosis	–0.01	7.6E–01	–0.02	6.9E–01	–0.02	6.8E–01	6.8E–01	474 (59)	26 (8)	86 (8)	0.05	0.45 (0.14)	5.82	42.60
Parkinson’s disease	–0.19	**2.0E–04**	–0.10	1.5E–01	–0.15	**1.7E–03**	**4.4E–03**	432 (58)	68 (15)	844 (126)	0.14	**–0.75 (0.14)**	1.55	41.25
Alzheimer’s disease	–0.06	4.3E–01	–0.22	5.3E–02	–0.13	9.2E–02	1.3E–01	485 (60)	15 (8)	52 (10)	0.03	**–0.61 (0.24)**	10.90	37.84
General cognitive ability	0.07	3.2E–02	0.12	**3.7E–03**	0.09	**1.1E–03**	**3.6E–03**	217 (70)	283 (51)	10,802 (358)	**0.57**	**0.93 (0.12)**	1.47	8.73
Educational attainment	0.10	**6.0E–04**	0.10	1.1E–02	0.10	**9.9E–05**	**4.3E–04**	166 (60)	334 (34)	12,855 (285)	**0.67**	**0.90 (0.11)**	2.41	9.45

­
