# Supplementary material for: Estimating bone marrow adiposity from head MRI and identifying its genetic architecture
Source: eLife. 2026 Nov 4;13:RP101499. doi: 10.7554/eLife.101499 (PMC13734677; doi:10.7554/eLife.101499)
Supplement: Peer review history

# Editor report: eLife Assessment

**DOI:** 10.7554/eLife.101499.3.sa0

**Authors:** Clifford J Rosen

**Affiliations:** Maine Medical Center Research Institute United States

**Keywords:** Convincing, Important

This study introduces an artificial-intelligence tool that estimates fat in skull bone marrow from routine brain scans, enabling large studies that were previously impractical. The evidence for the method's repeatability and for identifying genetic links is **convincing** overall. The authors identify genes, diseases, and other biological characteristics that are linked to skull marrow fat, which represents an **important** advance. The work will be of most interest to researchers using large imaging biobanks and those studying ageing-related changes across bone, blood, and brain.

# Referee report: Reviewer #1 (Public review)

**DOI:** 10.7554/eLife.101499.3.sa1

The authors of this study developed a method to quantify calvarial bone marrow from MRI head scans, enabling study of its composition in large datasets of adults, usually collected to study the brain. Bone marrow intensity can be semi-quantitatively measured in T1-weighted MRI scans due to the greater signal intensity of fat than watery red marrow. This is an ingenious use of the MRI-produced information for other important phenotypes, such as bone structure and marrow content. Different head types were tested for complying to the model, which is notable.

The model was also successfully validated using several publicly available MRI resources - real data - in (1) dataset consisting of 30 individuals that were scanned 10 times each at 3-day intervals, and (2) the monozygotic (MZ) twin data from the Human Connectome Project cohort. Then the authors applied this validated method to head-MRI scans from the UK Biobank (n=33,042) to extract information on spatial distribution of bone marrow adiposity (BMA) in the calvaria, allowing a GWAS to identify associated genes.

The authors revealed high heritability and identified 41 genetic loci significantly associated with the BMA trait, including six sex-specific loci. Of note, statistics estimate that 99% of BMA trait-influencing variants are shared with BMD (497 of 500 variants), which may mean these results demonstrate the biological relevance to bone health. Some of the BMA genes were found related to the Wnt pathway, including WNT16, WNT4, NXN; this is a "positive control", since the Wnt/β-catenin signaling pathway was suggested as an important determinant of BMA. Also, associations in genes (BMP4, DLX5, LGR4, LRP4, SFRP4) that are known to specifically influence adiposity, are encouraging. Integrating mapped genes with bone marrow single-cell RNA-seq data revealed patterns of adipogenic lineage differentiation and lipid loading.

The study also investigated genetic overlap between BMA and twelve (or 13) "brain and body" traits, and identified significant genetic correlations with BMI, cognitive ability and Parkinson's disease.

In sum, since MRI head scans present a hitherto unexplored opportunity to address unresolved aspects of bone marrow biology, this study is both timely and innovative.

Comments on revised version:

The authors responded most of this reviewer's comments. Their explanations are convincing. Yet, upon re-reading the revised version of this paper, I still have concerns about the clarity of mostly analysis presentation, e.g.:

Line 130-133: the sentence is still unclear: "To obtain the BM signal intensity for an individual datapoint of the calvarium, we ... averaged these BM intensities to get the (average?) BM intensity for that datapoint. Then, we averaged (again?) these datapoint intensities across the calvarium to produce the global BMA measure for the scan."

Also, I still cannot understand whether the "overlap between the true and predicted bone marrow ...below 0.7" is concerning or not, - whether this threshold of 0.7 is arbitrary.

Genetic correlation: pls. make sure it's clear that the Rg was calculated using SNP "effect sizes".

# Referee report: Reviewer #2 (Public review)

**DOI:** 10.7554/eLife.101499.3.sa2

Summary:

The authors set out to enable large-scale measurement of fat in skull bone marrow using routine structural brain MRI scans. They present a neural-network pipeline trained largely on realistic simulated examples and show that the resulting skull marrow measure is highly repeatable in test-retest data and consistent in monozygotic twins. Applying it to ~33,000 UK Biobank participants, they report expected population patterns (including sex- and menopause-related differences) and identify genetic and health-related associations, creating an important resource that can be built upon by researchers interested in BMA, imaging, bone, metabolism, neuroscience, ageing, haematology, and other fields.

Major strengths:

A notable methodological strength is the training strategy: by using a large, simulated dataset that captures plausible variation in skull-layer thickness and MRI intensity, the authors reduce reliance on scarce expert-labelled images. The modelling choice (using 1D intensity profiles through the skull rather than analysing the full 3D volume) appears well matched to the anatomy and offers an efficient approach for thin, layered structures. Multiple validation steps (including test-retest reliability and twin concordance) support the robustness of the measurement pipeline.

On the biological and genetic side, the study demonstrates that the skull BMA estimate relates to known correlates of marrow fat (e.g., age/sex/menopause patterns/bone density) and integrates population imaging with large-scale genetic analysis to highlight loci and candidate genes with plausible relevance to skeletal and marrow biology. The inclusion of cross-ancestry analyses and integration with cell-type-resolved gene-expression resources further improves interpretability and usability for the community.

Major limitations:

The main limitation is conceptual rather than technical: the phenotype is derived from T1-weighted MRI intensity, which does not directly separate fat and water signals and can vary with scanner and sequence settings. The manuscript provides convincing evidence that the measure is reproducible and biologically meaningful, but it should still be interpreted as a semi-quantitative proxy for marrow fat rather than a direct fat-fraction measurement. Accordingly, the genetic and phenotypic associations are likely informative, but the most direct claims about "adiposity" would be stronger if anchored to established quantitative fat-measurement imaging or spectroscopy in the skull.

The genetic "replication" analysis in a smaller, ancestrally heterogeneous non-European-ancestry sample is useful as a test of transferability, but it is not equivalent to replication in an independent cohort of similar ancestry and is expected to show reduced SNP-level reproducibility because of differences in sample size and genetic background. This should be clearly framed so readers understand what level of generalisation is supported by the current evidence.

Likely impact and utility:

Overall, the work provides a practical method for extracting new biological information from widely available brain MRI scans and should be particularly useful to researchers working with large imaging biobanks and those studying connections between bone, blood, metabolism, and brain ageing. The combination of a scalable measurement approach and openly reported genetic results is likely to accelerate follow-up studies, including cross-cohort comparisons and mechanistic work on candidate pathways.

# Referee report: Reviewer #3 (Public review)

**DOI:** 10.7554/eLife.101499.3.sa3

Summary:

This paper addresses a fundamental gap in bone biology: our near-complete ignorance of the in vivo dynamics of calvarial bone marrow adiposity (BMA) at population scale. The authors developed an elegant artificial neural network trained on simulated data to automatically localize and quantify the bone marrow layer within standard T1-weighted MRI head scans; scans originally acquired to study the brain but harboring rich, unexploited information about adjacent bone. Applying this method to over 33,000 individuals from the UK Biobank, they accomplished three things that had never been done before: (1) they precisely quantified the sex-dimorphic age trajectory of calvarial BMA, including the dramatic post-menopausal rise and the protective role of hormone replacement therapy; (2) they performed the first well-powered GWAS of this trait, identifying 41 genome-wide significant loci including six sex-specific ones, with SNP heritability of 31.5%; and (3) they revealed significant genetic correlations and overlap between BMA and traits including bone mineral density, Parkinson's disease, and general cognitive ability, a finding made all the more intriguing by the recently described direct vascular channels connecting calvarial bone marrow to the meninges. Integration of GWAS genes with single-cell RNA-sequencing data from mesenchymal lineage cells further illuminated which genes govern lineage commitment to the adipogenic pathway versus lipid loading in mature adipocytes.

Comments on revised version.

The reviews raised substantive points across three domains, and the authors engaged with every one of them seriously and thoroughly.

On the validation of T1-weighted MRI as a measure of BMA: Reviewer 2 raised the strongest concern, arguing that T1-weighted signal intensity had never been formally validated as a quantitative fat-fraction measure in the calvarium. The authors responded with both a principled scientific argument and new data. They assembled existing literature demonstrating that T1-weighted signal is an established semi-quantitative proxy for marrow fat in multiple skeletal sites (Loevner et al. 2002, Shen et al. 2013, Zhang et al. 2020), and provided additional comparative analyses against quantitative T1 relaxation maps, multiple intensity normalization strategies (KDE, WhiteStripe, GMM, FCM, Z-score), DEXA-derived bone mineral density, and osteoporosis status. The biological coherence of their findings, recapitulating known sex and age profiles, identifying genes already established in cell and animal models of BMA biology, and estimating heritabilities consistent with twin data constitutes powerful, convergent evidence for construct validity. Their point that a semi-quantitative measure of a highly variable, well-demarcated biological signal can outperform a perfectly precise measure of a poorly defined entity is methodologically sound and well-argued.

On sex differences and the role of Hyperostosis frontalis interna: Reviewer 1 raised the clinically astute concern that Hyperostosis frontalis interna (HFI), a condition of inner table thickening prevalent in up to 49% of postmenopausal women, could confound calvarial BMA measurements and drive apparent sex differences. The authors performed a dedicated new analysis, stratifying BMA-BMD associations by sex and age group. They demonstrated that (1) the BMA-BMD association is robust in both males and females, (2) it remains stable across age groups, and (3) the neural network trained on simulations incorporating wide anatomical variation including inner table thickness is inherently resistant to moderate inner table thickening. Given that HFI is restricted to the frontal bone, which represents only a fraction of the calvarial surface, and that severe cases are rare (ICD-10 prevalence ~0.02% in the UK Biobank), the authors make a convincing case that this does not materially bias their results. Their suggestion that the method could itself be used in future work to study the genetic architecture of HFI is a nice forward-looking addition.

On genetic correlation interpretation and cross-trait pleiotropy: Reviewer 1 asked for clarification of the vertical versus horizontal pleiotropy distinction and for formal Mendelian randomization to support the possible causal effect of BMA on cognition. The authors appropriately clarified the conceptual framework in the revised text and, rather than overstating a causal claim without the supporting analysis, responsibly softened the language to "may be consistent with the hypothesis that BMA could have a causal effect on cognition." This is scientifically honest and appropriate.

On mouse scRNAseq and its relevance to humans: The authors acknowledged that the results section had not explicitly stated the mouse origin of the scRNAseq data, corrected this, and provided a well-justified rationale for the relevance of mouse mesenchymal lineage data to human BMA biology, which is a well-established and widely accepted model system in this field.

On GWAS replication: The claim that the study lacked replication was addressed by clarifying the a *priori* separation of discovery (white British, n=33,042) and replication (non-white British, n=4,958) samples, with 62% of significant discovery SNPs and 95% of lead SNPs replicating in the correct direction.

Overall Assessment:

This is a technically innovative, scientifically rigorous, and biologically meaningful paper. The method is genuinely novel, the sample size is among the largest ever applied to this phenotype, the genetic findings are well-powered and well-replicated, and the integration across imaging, genetics, and single-cell transcriptomics is exemplary. The authors have engaged with every substantive reviewer criticism in good faith, producing new analyses where appropriate and defending, and convincingly, with findings that were challenged without adequate basis. The revised manuscript is strengthened throughout.

This paper opens a new window quite literally, through the skull - into bone marrow biology at a scale and resolution that has never been achieved before.

# Author comment: Author response

**DOI:** 10.7554/eLife.101499.3.sa4

**Authors:** Tobias Kaufmann, Pål Marius Bjørnstad, Martin Falck, Stener Nerland, Kevin O'Connell, Oleksandr Frei, Ole A Andreassen, Lars T Westlye, Srdjan Djurovic, Timothy Hughes

**Affiliations:** University of Tübingen Tübingen Germany; Oslo University Hospital Oslo Norway; Oslo University Hospital Oslo Norway; University of Oslo Oslo Norway; NORMENT, Institute of Clinical Medicine, University of Oslo, and Division of Mental Health and Addiction, Oslo University Hospital, Oslo, Norway Oslo Norway; University of Oslo Oslo Norway; Oslo University Oslo Norway; University of Oslo Oslo Norway; Oslo University Hospital Oslo Norway; Oslo University Hospital Oslo Norway

The following is the authors’ response to the original reviews.

**Public Reviews:**

**Reviewer #1 (Public review):**

The authors of this study developed a method to quantify calvarial bone marrow from MRI head scans, enabling the study of its composition in large datasets of adults, usually collected to study the brain. Bone marrow intensity can be semi-quantitatively measured in T1-weighted MRI scans due to the greater signal intensity of fat than watery red marrow. This is an ingenious use of the MRI-produced information for other important phenotypes, such as bone structure and marrow content. Different head types were tested for complying with the model, which is notable.

The model was also successfully validated using several publicly available MRI resources - real data - in (1) a dataset consisting of 30 individuals that were scanned 10 times each at 3-day intervals, and (2) the monozygotic (MZ) twin data from the Human Connectome Project cohort. Then the authors applied this validated method to head-MRI scans from the UK Biobank (n=33,042) to extract information on the spatial distribution of bone marrow adiposity (BMA) in the calvaria, allowing a GWAS to identify associated genes.

The authors revealed high heritability and identified 41 genetic loci significantly associated with the BMA trait, including six sex-specific loci. Of note, statistics estimate that 99% of BMA trait-influencing variants are shared with BMD (497 of 500 variants), which may mean these results demonstrate the biological relevance to bone health. Some of the BMA genes were found related to the Wnt pathway, including WNT16, WNT4, NXN; this is a "positive control", since the Wnt/β-catenin signaling pathway was suggested as an important determinant of BMA. Also, associations in genes (BMP4, DLX5, LGR4, LRP4, SFRP4) that are known to specifically influence adiposity, are encouraging. Integrating mapped genes with bone marrow single-cell RNA-seq data revealed patterns of adipogenic lineage differentiation and lipid loading.

With regards to the reviewer’s comment on the overlap between BMA and BMD trait-influencing variants, we would like to add that the correlation of effect sizes within the overlap is -0.95 as we would expect from bifurcating differentiation of mesenchymal stem cells into osteoblasts or adipocytes: the underlying common biology driving these traits results in shared traits with a negative correlation in the effects.

The study also investigated the genetic overlap between BMA and twelve (or 13) "brain and body" traits and identified significant genetic correlations with BMI, cognitive ability, and Parkinson's disease.

In sum, since MRI head scans present a hitherto unexplored opportunity to address unresolved aspects of bone marrow biology, this study is both timely and innovative.

There are, however, some assumptions, findings, and their interpretation, which require more critical focus.

Sex-specificity is well described and studied here. Men have higher BMA than women, but post-menopausal women catch up in the BMA values. The authors believe that calvarial marrow has a number of features that make it particularly well-suited to the study of BMA process - which is clinically important in other bone sites. It has a simple "sandwiched" structure that they are able to model. This is true only to some extent: a condition called "Hyperostosis frontalis interna", of unknown etiology (described by Smith & Hemphill in 1956) - is characterized by irregular overgrowth of the inner table of the frontal bone (symmetric/bilateral). Although not of clinical significance, typically benign, studies report a prevalence of 12%; However, it's most common in postmenopausal women - where prevalences up to 49% in women over the age of 65 - have been reported. Thus, sexual dimorphism is obvious and the effect of estrogen is likely shared with whichever bone - and marrow - age-related pathology. So, for women not using HRT, this new layer of the bone might interfere with the calvarial BMA readings and in turn, affect the BMA-related analyses.

Thank you for bringing the "Hyperostosis frontalis interna" condition to our attention. It is particularly interesting to hear that the etiology is unknown and one may suspect that some kind of calvarial bone marrow dysregulation may be part of the cause. Our model for bone marrow location was trained on simulated data which included variation in the thickness of all anatomical layers (including the inner table), so it will be robust to some thickening of the inner table. It might not be robust to the most extreme cases of inner table thickening (as described in some case reports), but these are rare. Further, it should also be noted that the other calvarial bones, representing a much greater fraction of the calvarial surface, remain largely unaffected by the thickening and would therefore yield correct localisation of the bone marrow layers. In summary, although the severe cases of hyperostosis frontalis interna have the potential to affect our identification of the bone marrow layer, the low frequency of such cases and the restriction of the phenotype to the frontal bone means that the potential for bias is very limited.

It would be interesting to develop a method for detection of thickened inner bone so that the condition’s prevalence can be quantified in a large sample like the UK Biobank and its genetic architecture be determined. This could help elucidate the etiology.

The authors suspect that the effect of BMA on BMD may be biased in women; they should comment on those "with low BMD and high BMA" given that hyperostosis frontalis might be an issue. A strong effect of SNPs in the ESR1 chromosomal region might be akin to the above concern.

Thank you for raising this point, which we have followed up with a new analysis.

According to ICD-10 data in UK Biobank there are only N=105 individuals with an M85.2-diagnosed disorder. Given the total sample size of N=446,814 individuals with ICD-10 data, this would translate to a prevalence of 0.02%, which speaks for an underdiagnosis in this sample such that we cannot simply remove diagnosed individuals to control for a potential diagnostic confound.

We have therefore taken a different approach to investigate this potential issue: As you elaborated in your previous comment, the prevalence of hyperostosis frontalis increases with age in females. The literature also suggests that prevalence rates do not differ between males and females in young age / prior to menopause. Therefore, we have studied the association between BMD and BMA for males and females separately, and in two age groups based on a median split of our sample (left plot: younger than 65, right plot: subjects older than 65). In these plots, the relatively large shift in female BMA and BMD is visible with the large yellow cloud at low BMA and high BMD in the left plot disappearing in the right plot. Despite this, we observe:

(1) Associations in both males (blue) and females (yellow), suggesting that the associations were not driven only by females.

(2) BMA-BMD association is largely similar across the two age groups.

If we consider that the old age group is likely to contain more cases of hyperostosis frontalis than the young group, and if we consider that old-aged females are more likely to be in this condition than men of any age, then we would expect an impact of hyperostosis frontalis on our measures to result in observable differences in Author response image 1. This is not the case. We see global age-related shifts in BMA in women, yet the association with BMD remains similar across age groups.

The technical properties of our neural network (trained on simulated data) makes it unlikely that frontal bone will contaminate the bone marrow detection globally (description above) and these results show that hyperostosis frontalis is not a considerable issue in our analysis.

**Author response image 1.**

Then, there is a perfect overlap of the BMA SNPs that are shared with BMD (497 of 500 variants), which may prove a "face validity" of the MRI-derived BMA. However, the BMD in the study was heel-derived eBMD - which is a good proxy for osteoporosis and is mostly driven by trabecular bone. Thus, there might be a concern that the BMA metrics capture some trabecular BMD.

The reviewer is correct in pointing out that the BMA causal variants are a near-perfect subset of the BMD causal variants. The reviewer raises the concern that the BMA measurements may capture some trabecular BMD, however it should be noted that the correlation of effect sizes for the BMA/BMD overlapping causal SNPs is negative (-0.95). If our measure of BMA had been erroneously capturing trabecular BMD then we would expect to see a positive correlation of effect sizes for the BMA/BMD overlapping causal SNPs, not a negative one.

Next, integrating mapped genes with existing bone marrow single-cell RNA-sequencing data revealed patterns of adipogenic lineage differentiation and lipid loading. The problem here is that the scRNAseq studies of the Bone Marrow niche are overwhelmingly mouse. The authors might wish to justify why they are relevant to humans (in the absence of the human-specific scRNAseq).

We thank the reviewer for pointing this out. We noticed that, although Figure 4 and the Methods do explicitly state that the scRNAseq data is from mouse, it is not stated in the text of the Results. This is now corrected.

The mouse is commonly used as the model organism for in vivo investigation of human phenotypes and bone marrow adiposity is no exception because, although mice have lower bone marrow adiposity than humans, the timing and sequence in bone marrow adiposity development are similar. BMA research makes extensive use of mouse models literature as exemplified by this review of research within the field (here) and this article recent article (Koh et al. 2024. “Adult skull bone marrow is an expanding and resilient haematopoietic reservoir”. https://www.nature.com/articles/s41586-024-08163-9)

We updated the results section (line 279):

“Mesenchymal stem cells of the BM niche commit to either the adipogenic or the osteogenic lineage (Figure 4A) and both the number committing to the adipogenic lineage and their level of lipid-loading influences the total level of BMA. This aspect of BM biology is shared between humans and mice (29), so we made use of an existing mouse scRNAseq dataset of BM mesenchymal lineage cells (30) to study variation in the expression of BMA-associated genes as cells differentiate (Figure 4B).”

For genetic correlation analysis, the authors selected 7 body and 6 brain traits. The latter traits reflect cognition (general cognitive ability and educational attainment) and brain-related disorders. This selection might seem arbitrary. The interpretation of genetic correlation with cognitive ability, education, and Parkinson's disease was attributed to the recently discovered vascular channels that link calvarial bone marrow to the meninges. This is a fascinating hypothesis, which requires functional proof. However, there might be simpler explanations. Thus, the diploe and the inner table of the calvarium are drained by the same veins as the dura. From the anatomy textbook, we know that diploic veins connect the pericranial and endocranial venous system through the skull.

Whilst it is true that we did not systematically compare the results of the BMA GWAS to all potentially relevant brain and body phenotypes, we did use criteria to select the phenotypes we compared to. As stated in the manuscript (line 304):

“We selected body traits (BMD, BMI, waist-to-hip ratio, systolic and diastolic blood pressure, type-2 diabetes, coronary artery disease) that have a logical connection to BMA given the mesenchymal stem cells origin of BM adipocytes and their role in bone, fat, and vasculature (29). For the brain, we selected traits reflecting cognition (general cognitive ability and educational attainment) and disorders that are prevalent in adulthood (insomnia, multiple sclerosis, Parkinson's disease, Alzheimer's disease) since it is primarily in adulthood that the adiposity of calvarial BM experiences a substantial change”

We entirely agree that the suggestion that the genetic correlation between BMA and cerebral traits may be mediated by the vascular channels linking calvarial bone marrow to the meninges is merely a hypothesis. We have therefore updated the text of the Discussion (line 470):

“We tentatively speculate that calvarial MALPs may be involved in sensing perivascular flows of CSF from the meninges to the BM and in influencing the BM’s hematopoietic response, and that this might be the basis of the observed genetic overlap between BMA and some cerebral traits. However, more conventional anatomical pathways may also be relevant, as the diploë and inner table communicate with meningeal and dural venous systems through diploic veins.”

**Reviewer #2 (Public review):**

Summary:

This study develops a new artificial intelligence method for high-throughput analysis of skull bone marrow from MRI data, which may be useful for large-scale biological analyses. Using this method, the authors then attempt to estimate skull bone marrow adiposity (BMA) using T1-weighted signal intensity from MRI scans of ~33,000 people, followed by genome-wide association analysis; however, the approach is inadequate because T1-weighted signal intensity is not validated for measurement of bone marrow adiposity. If it could be validated, the study would be an important advance in understanding of bone marrow adiposity and skeletal biology.

Strengths:

This paper is well-written, and the figures are nicely presented. The neural network method used for analysing skull bone marrow is innovative, and the authors validate this through several approaches. Therefore, the authors have achieved the aim of developing a method for large-scale analysis of skull bone marrow from MRI data.

The GWAS is reasonably well-powered and addresses potential ethnicity differences, with one GWAS done across white males and females, and a separate GWAS in non-white participants. The methodology also conforms to common GWAS standards, including for mapping genetic variants to candidate genes. Moreover, the study further investigates the biological roles of these genes by analysing their expression in single-cell RNA sequencing data.

Weaknesses:

The fundamental weakness is that T1-weighted MRI signal intensity (T1W) is used as an estimate of BMA, but it has never been validated for this. The authors show that this T1W parameter measures something that is heritable and can be compared between subjects, but they don't show that it actually measures (or even estimates) calvarial BMA. There is an attempt to do so by comparing the T1W parameter with data from quantitative T1 images: the authors show a reasonable correlation with some of the quantitative T1 image data. However, this still does not show that the parameter is measuring BMA; it could be measuring some other biological characteristic, but this remains unclear. So, there is a need to validate the T1W parameter against an established measure of BMA, such as the bone marrow fat-fraction or proton density fat fraction measured from multi-echo MRI analysis.

Without validating this BMA measurement method, it is not possible to interpret the GWAS or other findings reported in the study.

We reject this criticism.

Although T1-weighted has not been validated as a quantitative measure of fat-fraction, there are several studies showing that it is a semi-quantitative measure of fat content (e.g. Loevner et al 2002, Shen et al 2013, Zhang et al 2020) and we also provide data that support this (figures S9-11).

Semi-quantitative measures are used in many biomedical GWASes for instance even highly heritable neuropsychiatric disorders (such as schizophrenia and bipolar disorder) involve assessment by clinicians where the test-retest kappas are in the range 0.4-0.6.

Further, we would suggest that the shortcoming of the imperfect correlation of T1w signal intensity with fat content is more than outweighed by our precision in identifying the calvarial BM cavity and the fact that the flat calvarial bone marrow has a wide range of adiposity in middle-aged and elderly individuals (compared to other bones). This lies at the root of why:

We clearly recapitulate the known sex and age profiles, as well as the effect of HRT.

We estimate high BMA heritabilities (43% in males and 23% in females)

We find clear sex differences (which is a known feature of BMA biology)

We identify a large number of the genes already known to affect BMA from earlier animal and cell work

A noisy measurement of an entity with strong biological signal (a well-defined bone marrow cavity with variation in BMA across subjects) will often be more informative than a highly precise measurement of a poorly defined entity with little signal.

A less critical weakness is that the GWAS has been done only on a single cohort, without replicating the findings in a follow-up cohort. For example, the authors could repeat their analysis on the remaining ~50,000 UK Biobank imaging participants for whom MRI data is now available. However, this would be pointless without knowing what biological characteristic(s) the T1W parameter is actually reflecting.

We disagree with this comment. We separated the UKB data into discovery and replication sets prior to running the GWAS, so these datasets are independent:

(1) Further, we ran the discovery (white british individuals) GWAS separately for males and females (prior to combining) and reported in the results section: “We found them to have low genomic inflation (Figure 3A and Table S4) and to be significantly genetically correlated (Rg=.94, P=6e-27, Figure 3B)”

(2) We performed our replication GWAS in non-white British males and females. As noted in the results section: “Out of the 168 significant discovery SNPs, 62% replicated at P<.05, and 39% of the 41 lead SNPs replicated at P<.05 (Table S6). One locus replicated at genome-wide significance (P<5e-8). Furthermore, 92.7% of the lead SNPs of the discovery sample showed same effect direction in the replication sample (Table S5).”

**Reviewer #3 (Public review):**

Summary:

This manuscript, "Estimating bone marrow adiposity from head MRI and identifying its genetic 2 architecture", brings together the groups of Drs. Kaufmann and Hughes in a tour de force work to develop an artificial neural network that localizes calvaria bone marrow in T1-weighted MRI head scans, with the goal of studying its composition in several large MRI datasets, and to model sex-dimorphic age trajectories, including the effect of menopause.

Strengths:

Bone marrow adiposity is a very active tissue with far-reaching implications for tissue crosstalk and human health than we had initially recognized. Although MRI has been used to measure BM, studies such as the one by these two groups are still lacking whereas very large datasets are analyzed using advanced AI machine learning tools coupled with genetic studies and a specific pathology. The groups had to develop new methods and new AI machine-learning tools for the imaging analyses.

Weaknesses:

Some aspects of the work that authors could add additional clarification.

(1) Imaging Limitations: The authors provide an excellent overview and references supporting the use of MRI as a method for assessing marrow fat, particularly with some specific modifications. However, MRI images can be affected by various factors, including the presence of other tissues as well as specific MRI settings, which are much harder to precisely control when using different datasets.

We thank the reviewer for his positive assessment of our review of methods.

Regarding MRI settings: We agree with the reviewer that differences in scan protocols can create substantial differences in the resulting images between samples. Different tools exist for harmonization of imaging data across sites, but they usually operate on tabulated data and there is no one-size-fits-all approach yet [1]. Here, we took a different approach to prevent confounding bias: We generated a large set of simulated data for training of the neural network. The simulations circumvented potential issues emerging from confound biases in training sets that we might have seen had we had combined multiple samples with different scan protocols. Nevertheless, applied to real data the models may still face confound issues, such as better BMA estimates for some scan protocols over others. We have addressed these issues as follows: (1) Validation analyses (10 repeat scans of 30 individuals and twin pairs, figure 1d and 1e) are based fully on data that was acquired on the same scanner with the same protocol. (2) Analysis in UK Biobank included data from different scan sites albeit harmonized protocols. Here we accounted for scan site in all statistical models (including GWAS).

(1) Dominik Kraft, Gloria Matte Bon, Édith Breton, Philipp Seidel, Tobias Kaufmann; Removing scanner effects with a multivariate latent approach: A RELIEF for the ABCD imaging data?. Imaging Neuroscience 2024; 2 1–7. doi: https://doi.org/10.1162/imag_a_00157

Regarding the presence of other tissues: We recognise in the existing text of the results section that sometimes inner or outer table voxels are wrongly identified as bone marrow, but we show that this does not have a major impact on the correct identification of the bone marrow cavity. The existing text reads:

“Poor overlap (below 0.7) was almost only observed in the thinnest bone and is explained by the fact that when the BM part of the bone is only a few layers thick (1 layer = 0.5 mm), an error by one layer will inevitably lead to a substantial fall in overlap. However, this did not result in a corresponding fall in the ratio of the predicted intensity of BM to its true intensity, because the typical BM intensity was only marginally higher than the neighbouring bone intensity. This property of the typical relative intensities of these anatomic structures also explains why the intensity ratio at high overlap is not centred on 1: any misidentification of cortical bone as BM, will typically result in an underestimate of true BM intensity (Figure S2). The neural network performed well and intensity ratios were in the range 0.9-1.1 for the vast majority of head types (Figure S3).”

Also note that we implement a number of QC measures to exclude scans where there is evidence that we may have failed to correctly identify the bone marrow cavity. The existing text reads:

“We used two additional QC metrics to filter out calvaria where BM location was likely to have failed. First, we set an upper limit of 30 on the standard deviation of the intensity of the outer table as scans with higher values were clear outliers and were probably cases where the location of both outer table and BM has failed (Figure S6). Second, for each calvarium, we computed the Mahalonobis distance for all vertices in the two dimensions “first layer of the BM” and “BM intensity” (Figure S7). By manual inspection we found that data points with MD > 25 often had errors in BM layer identification, typically where the network had erroneously predicted a higher and more intense layer to be the BM. We considered a calvarium as failing this QC criterium if more than 0.5% of vertices have MD > 25. This criterium is very strict as errors on only 0.5% of data points in a calvarium would not significantly affect the average BM intensity for a calvarium.”

(2) The specific density of cranial bones as it relates to the types of bone marrow: Cranial bones are extremely dense structures, which naturally interfere with MRI imaging. While it is thought that cranial bones have mostly "red bone marrow", this is only true for a short time in humans. How sensitive is their system in differentiating between red and yellow BM?

We implemented several measures to ensure that our method would be robust to anatomical variation between individuals. As noted in the current version of the Methods section: “In order to train the neural network model, we generated a large synthetic dataset of intensity arrays, with known boundaries between anatomical structures, by simulating the thickness and intensity of the different structures located between the outer skin and the subarachnoid space. The simulation incorporated the following real-world complexities:

Different anatomical architectures (skin, subcutaneous fat, aponeurosis, outer table, BM, inner table, dura mater, arachnoid space), including when a structure is not present throughout the calvarium

Variation in thickness and intensity between vertices (on the same calvarium)

A wide variety of different calvarium types with different combinations of levels of BM adiposity, bone thickness, and subcutaneous adiposity.”

Further, as noted in the Results section:

“We evaluated the performance of the neural network on simulated data using two metrics (Figure 1B): 1. the overlap between the predicted and the true BM location, and 2. the ratio between the predicted intensity of the BM and the true intensity of the BM”. The accuracy in localising the bone marrow layers was good, with the only exception being: “Poor overlap (below 0.7) was almost only observed in the thinnest bone and is explained by the fact that when the BM part of the bone is only a few layers thick (1 layer = 0.5 mm), an error by one layer will inevitably lead to a substantial fall in overlap”

We also validated our procedure on real data (see Results section, subsection “Procedure validation on real data and heritability estimate”). Briefly, we checked the accuracy of our method using a dataset from the Consortium for Reliability and Reproducibility, a twin dataset from the Human Connectome Project and by manually checking many hundreds of UKBiobank scans.

We are thus confident that we accurately identify the bone marrow cavity irrespective of whether the bone marrow is red (low adiposity) or yellow (high adiposity).

(3) Both items above are further complicated by aging, but aging is not a linear event as we have learned. There are specific bursts of aging in humans around the age of 45 and early 60s. How do the system and model predict or incorporate these peaks of aging? It seems from the data shown that aging is reflected more as a linear phenomenon. Is this because additional aging datasets are needed?

We agree with the reviewer that ageing probably occurs in bursts rather than being a linear process. We do see a non-linear relationship between age and BMA in our data (see figure 2B), with a more rapid rise in BMA between the ages of 45 and 65, than later in life (in women). As a result of this, when we model BMA using regression, we use orthogonal polynomials of degree 2 which allows for a non-linear relationship. However, we cannot observe bursts of BMA increase in our data because it is cross-sectional. Longitudinal data would be required to obtain information on the nature and timing of any bursts in bone marrow adiposity.

(4) The authors describe in richness of detail their AI learning programming and how it extracted the data from datasets. The authors also show some important correlations with specific genes, SNPs. What is not clear is how conditions such as anemia for example. An expected finding would be that patients with chronic anemia have lower bone marrow (BM) signal intensity on MRI scans than healthy people. This is because the signal intensity of BM depends on the fat-to-cell ratio in the tissue.

We agree with the reviewer that conditions affecting the bone marrow niche have a potential to affect and be affected by bone marrow adiposity, with leukemia being a known example. This is why we believe that a method, such as the one we present here, has the potential to be useful in several biomedical fields (hematology and osteology).

Furthermore, patients with a host of musculoskeletal disorders ranging from osteopenia to osteoporosis, sarcopenia, and osteosarcopenia will also have altered MRI scans. When using such large datasets how did the authors control or exclude these pathological conditions, or were all these conditions likely present?

We did not exclude specific pathologies. We were careful to train our NN model on a wide variety of skull thicknesses, bone marrow adiposity, and subcutaneous adiposity and to evaluate the performance of the model on simulated and real datasets (see answer to your point 2).

Reviewer 1 raised the issue of individuals displaying Hyperostosis frontalis interna (thickening of the inner table) and we recognize that in extreme case of this condition, where there is a major change in the anatomy of the calvarial bone, our method would probably not correctly localise the bone marrow. However, such extreme cases are rare and thus would not have a major impact on our results derived from over thirty thousand individuals. We demonstrate this with an extra analysis performed in response to the point about hyperostosis frontalis interna made by reviewer 1.

(5) Some of the genes and SNPs although significant showed very small correlations. What is their likely physiological significance?

Bone marrow adiposity is a polygenic trait and we have identified 41 statistically significant loci. We had a discovery sample of approximately 30k individuals which is modest for a GWAS study, so these 41 loci are a lower bound on the number of genes influencing the BMA trait. When a large number of genes influence a trait, the effect size of an individual gene is typically relatively small. However, the SNP heritability estimates of 31.5% indicates that we are able to explain approximately one third of the phenotypic variation with the effect sizes estimated by our GWAS: this is quite a high fraction relative to many other GWASs of biomedical traits.

(6) The authors could use this excellent manuscript to expand their discussion to include the need for studies like theirs to be also complemented by multi-OMICS studies that will include proteomics and lipidomics of BM, bones, and muscles.

We agree with the reviewer and hope that such studies will be undertaken in the future. We attempted to point in this direction in the last sentence of the Discussion (line 517): “Future studies can build on our developments to further validate the proposed measure of bone marrow composition and to study its effect on bone, blood, and brain”. Word count limits prevented us from further expanding on the specific kinds of studies that should be performed.

**Recommendations for the authors:**

**Reviewer #1 (Recommendations for the authors):**

More moderate concerns include:

(1) In the "Genetic correlation and overlap" part, it is unclear why a high effect correlation with high SNP overlap is suggestive of vertical pleiotropy, while "moderate overlap and a low correlation of effect sizes ... are more indicative of horizontal pleiotropy". This is not intuitive.

In vertical pleiotropy (genetics > phenotype A > phenotype B): genetics drive phenotype A and phenotype A drives phenotype B (B has few direct genetic drivers of its own). If we perform a GWAS of phenotype A and a GWAS of phenotype B, one would expect to see a high overlap in the causal variants (because phenotype B is largely indirectly determined by the genetics of phenotype A) and the correlation should be high (either negative or positive) because there is a cause-effect relationship between A and B (whether phenotype A has a positive or negative effect on phenotype B).

In horizontal pleiotropy: the A and B phenotypes share the same genetic loci (but have no phenotypic influence on each other). In this case, we would observe high overlap in the associated loci, but one would not expect to see a high correlation in effect sizes (across loci) because there is no a *priori* reason to expect that genes associated with both phenotype A and phenotype B would have a consistent (negative or positive) effect across loci. For example, gene X may increase A and B, whereas gene Y may increase A but decrease B.

We have made a small update to the relevant part of the Results section and otherwise rely on the explanation above (which will be publicly available along with the manuscript):

Line 325: “These patterns of high overlap and high effect correlation within this overlap are suggestive of vertical pleiotropy i.e. a molecular mechanism influencing one trait, that in turn influences a second trait, such that most of the variants driving the first trait either have the same or the opposite direction of effect on the second trait.”

(2) "possible causal effect of BMA on cognition" asks for a formal analysis, like Mendelian randomization.

Given the current wording, the reviewer is justified in asking for a formal analysis. Since we did not perform this analysis, we have changed the wording:

Line 434: “Since these are two highly correlated traits [36], a high overlap and correlation of genetic effects for both traits with BMA may be consistent with the hypothesis that BMA could have a causal effect on cognition (Table 1).”

(3) ll. 132-134: please reword this sentence for clarity: "Using the network-estimated location of the BM within ... averaged these across all datapoints...". Please define threshold of desirable overlap between the predicted BM and the true BM (=0.7?).

Background: The model predicts the BM localisation for a datapoint (which interval of layers of the 50 layers is bone marrow). We tested the model on a wide variety of simulated data and aim for the overlap to be as close to 1 as possible, but some error is inevitable. We found that the average overlap between the true and predicted bone marrow was only below 0.7 when the bone layer is only 4 mm thick (meaning that the bone marrow is only 1-2 mm thick). This demonstrates the high accuracy of our method in identifying a very small anatomical feature.

When applying our method to real data, we do not know the truth and therefore cannot compute the overlap between the predicted and true value. It is therefore not possible to identify datapoints where the overlap is poor (e.g. lower than 0.7) and filter them out.

Given the above, we struggle to understand in what way an overlap threshold is relevant to how we compute the signal intensity for a datapoint. Nevertheless, we recognize that the sentence pointed to by the reviewer is poorly formulated and have tried to make it clearer:

Line 130-133: “To obtain the BM signal intensity for an individual datapoint of the calvarium, we used the network model to estimate the location of the BM within the datapoint’s intensity array and averaged these BM intensities to get the BM intensity for that datapoint. Then, we averaged these datapoint intensities across the calvarium to produce the global BMA measure for the scan.”

In the GWAS Results, please clarify the phrases - what was "significantly genetically correlated (Rg=.94)" (also, l. 402, "genetic correlation between the sexes" - in what?).

Genetic correlation is a statistical measure that quantifies the extent to which two traits (or the same trait in two different cohorts) are influenced by the same genetic factors. Simply put, it is the effect sizes of the SNPs in the two GWASs of interest that are correlated (after correcting for confounding effects, such as linkage desequilibrium). When comparing two GWASs, the standard formulation is to refer to their “genetic correlation”. We made a modification to the text to clarify this:

Line 239: “We found the male and female GWASs to have low genomic inflation (Figure 3A and Table S4) and to be significantly genetically correlated (Rg=.94, P=6e-27, Figure 3B)”

"a more than two-fold difference between the sexes" - in which metric?

We feel that what is being compared is stated clearly in the original sentence:

Line 262: “A comparison of the male and female effect sizes of the top lead SNPs of each locus revealed 6 loci in which there is a more than two-fold difference between the sexes (loci 10, 18, 26, 30, 32, 37 in Table S5)”.

(4) Also In GWAS Results, a locus Dlx5 is called "SHFM" in the Supplementary Table.

Background:

We identified 41 genome-wide significant loci and named the locus after the gene closest to the top lead SNP (bold in Figure 3C). Other genes in each locus for which genome-wide significant SNPs were eQTLs, are listed below the closest gene in normal font (Figure 3C).

In table S5, we report details of the top lead SNP for all 41 loci. We report only the nearest gene to the top lead SNP.

For locus 14, SHFM1 is the closest gene to the top lead SNP whereas DLX5 and DLX6 are genes in the locus for which genome-wide significant SNPs were eQTLs. This explains why DLX5 appears under SHFM1 in Figure 3C, but does not appear in Table S5.

(5) Please reword MRI jargon - "Dixon method", vertix - should be introduced, as well as abbreviation "KDE".

We had recognised that the word “vertex” would be confusing and had replaced it by datapoint, but had unfortunately missed one occurrence in the text. This is now corrected.

Thank you for pointing out the lack of introduction of the term “KDE”. This was only explained in the supplementary materials, but has now been added to the main text:

Line 500-508: “To ensure between-subject comparability, we used the intensity normalised nu.mgz volume output by FreeSurfer. We validated this approach through comparison with well-established intensity normalization methods; Kernel Density Estimation (KDE), WhiteStripe (WS), Gaussian Mixture Model (GMM), Fuzzy C-Means (FCM), and Z-score normalization (ZS). We found the highest test-retest reliability with our approach (Figure S9), and, together with KDE (based on reference signal intensity in WM), the highest correlation with quantitative T1 relaxation maps (Figure S10).”

**Reviewer #2 (Recommendations for the authors):**

(1) This would be an extremely useful advance for the bone and BMA fields if only it could be confirmed that the T1W signal intensity is actually measuring BMA in some meaningful way. Or, even if not BMA, to confirm what other biological characteristic(s) it is in fact capturing. This is essential for interpreting the findings.

(2) I note that you have compared the normalized T1W parameter with quantitative T1 data (e.g. Figure S10). However, this doesn't address the fundamental issue, because even these quantitative T1 data (e.g. from MP2RAGE) may not be measuring calvarial BMA. T1W sequences have been used to estimate BM cellularity (if not BMA directly) but are not nearly as precise as water-fat imaging. For example, one study found a reasonable correlation (0.71) between T1 relaxation times and BM fat (https://www.nature.com/articles/s41598-019-57030-5). So, if your normalized T1W parameter shows a correlation of -0.44 with the T1 MP2RAGE MRI signal (Figure S10), what does this mean in terms of how well your parameter reflects the actual BMA adiposity? We can't know this, because we also don't know if the T1 MP2RAGE signal reflects calvarial BMA.

(3) I think my recommendations are clear from the public review. Ideally, you would be able to compare the skull BM normalized T1W parameter with PDFF data that have T2* correction (since the skull BM cavity is quite small and so may suffer from T2* effects relating to tissue inhomogeneity). But even if you had only dual-echo BMFF data, this would still be much more informative than relying only on T1 data. I hope this can be done so that the findings of the study can be properly interpreted.

As explained above, we reject this reviewer’s claim that T1-weighted signal intensity cannot be used to perform a GWAS of BMA: other studies have shown that T1-weighted signal intensity is a semi-quantitative measure of fat fraction, we have performed extra analyses that confirm this, and our results further demonstrate this. For further detail on why we reject this criticism, see our response to this reviewer’s comments.
